# Enabling cell recovery from 3D cell culture microfluidic devices for tumour microenvironment biomarker profiling

**DOI:** 10.1038/s41598-019-42529-8

**Published:** 2019-04-17

**Authors:** María Virumbrales-Muñoz, Jose M. Ayuso, Alodia Lacueva, Teodora Randelovic, Megan K. Livingston, David J. Beebe, Sara Oliván, Desirée Pereboom, Manuel Doblare, Luis Fernández, Ignacio Ochoa

**Affiliations:** 1 Department of Biomedical Engineering, Wisconsin Institutes for Medical Research, University of Wisconsin-Madison, 1111 Highland Avenue, Madison, Wisconsin 53705 United States; 2 Medical Engineering, Morgridge Institute for Research, 330 N Orchard street, Madison, WI 53715 USA; 3 Group of Applied Mechanics and Bioengineering (AMB), Aragón Institute of Engineering Research (I3A), University of Zaragoza, Zaragoza, Spain; 4 Centro Investigacion Biomedica en Red. Bioingenieria, biomateriales y nanomedicina (CIBER-BBN), Madrid, Spain; 5 Aragon Institute for Health Research (IIS Aragón), Instituto de Salud Carlos III, Zaragoza, Spain; 6 Department of Chemistry, University of Wisconsin-Madison, Madison, USA; 7 Department of Pathology and Laboratory Medicine, University of Wisconsin-Madison, 1111 Highland Avenue, Madison, Wisconsin 53705 United States; 8 Servicio General de Apoyo a la Investigación de Citómica, University of Zaragoza, Zaragoza, Spain

**Keywords:** Cancer models, Biomedical engineering

## Abstract

The tumour microenvironment (TME) has recently drawn much attention due to its profound impact on tumour development, drug resistance and patient outcome. There is an increasing interest in new therapies that target the TME. Nonetheless, most established *in vitro* models fail to include essential cues of the TME. Microfluidics can be used to reproduce the TME *in vitro* and hence provide valuable insight on tumour evolution and drug sensitivity. However, microfluidics remains far from well-established mainstream molecular and cell biology methods. Therefore, we have developed a quick and straightforward collagenase-based enzymatic method to recover cells embedded in a 3D hydrogel in a microfluidic device with no impact on cell viability. We demonstrate the validity of this method on two different cell lines in a TME microfluidic model. Cells were successfully retrieved with high viability, and we characterised the different cell death mechanisms via AMNIS image cytometry in our model.

## Introduction

The tumour microenvironment (TME) is currently recognised as a central player in tumour development and progression^1^. TME comprises the niche in which a tumour develops and includes different cell types and distinctive cues^2^: gradients of oxygen, nutrients, and waste products are established in the malignant tissue, varying as a function of distance from the nearest supporting blood vessels to the centre of a tumour^3^. These specific TME cues are known to fuel tumour cell malignancy and tumour evolution, also having a profound impact on drug resistance and, as a consequence, on patient outcome^1,4^. Given the importance of the TME in tumour biology, the possibility of therapeutically targeting the TME has recently drawn considerable attention. In the last years, many different TME markers have been involved in tumour progression, neovascularisation, chances of metastasis and, patient prognosis. Among these, *Mki-67* (ki-67 protein) high expression has long been known to correlate with an exacerbated proliferation rate in the tumour site, hence forming a hostile TME^5^. The resulting environment leads to nutrient starvation, due to which cancer cells have been shown to activate alternative metabolic pathways to survive, resulting in an accelerated metabolic rate along with an elevated glucose uptake^6^.

Additionally, due to the high cell density inside the tumour mass, and the accelerated metabolism; an acidic pH is typically observed in the TME. Consequently, cancer cells activate different pathways to modulate their intracellular pH. Finally, tumour cells exhibit multiple survival mechanisms (e.g. stress responses) to endure the harsh and starving conditions generated within a tumour, allowing their escape from death mechanisms such as apoptosis and necroptosis^5,7^. All these cited factors can provide potential therapeutic opportunities for targets in the TME, since they promote a more hostile environment, and in turn worsen patient prognosis. Therefore, several approaches have been proposed in the literature to target the described TME cues and hence normalise the tissue microenvironment and eventually induce cancer cell death^8^.

Nevertheless, we still have an insufficient understanding of how to target these aspects of the TME efficiently. Potentially, one of the reasons for this is that reproducing the TME cues described above using *in vitro* traditional 2D cell culture methods based on the use of the Petri dish is exceptionally challenging. In this context, microfluidic-based platforms can reproduce complex biological three-dimensional microenvironments that mimic multiple aspects of the TME. Thanks to the small volumes manipulated through microfluidics and the physical properties of fluids at the microscale, spatial control can be achieved, and gradients can be utilised to create a three-dimensional biomimetic microenvironment^9,10^. These advantages have been previously used by many labs to develop biomimetic models of the tumour microenvironment^11–13^, including cues like the interaction among several compartmentalised cell types^14–18^, starvation^19^, chemotaxis^20–24^, mechanical stimuli^25,26^ and biochemical gradients^27–31^. Thus, complex scenarios inaccessible to traditional technologies can be investigated through microfluidics. Despite the advantages of microfluidics, the adoption of these techniques in mainstream biology research has not yet met the expectations surrounding the field. Arguably, the reason could be the gap existing between microfluidic techniques and other techniques found in traditional biomedical research^32^. In this context, most of the microfluidic assays only offer a low number of read-outs, generally based on microscopy observations (e.g., migration of cells towards chemoattractants or immunofluorescence). In contrast, an in-depth genomic or proteomic analysis remains extraordinarily challenging due to the high difficulty of retrieving cells in 3D culture from the microdevice.

In this work, we have taken advantage of the microfluidic TME model previously reported by our lab^31^ and further investigated processes related to tumour development through quantitative polymerase chain reaction (qPCR) and AMNIS image cytometry, a technique that provides simultaneously single-cell images and flow cytometry traditional analyses. More specifically, we have developed a method to retrieve cells from 3D collagen ECM scaffolds confined within microfluidic devices using a quick and straightforward enzymatic degradation process which does not affect cell viability. Although collagenase digestion has been already used for this purpose in the literature^33–35^, very little detail is provided on the procedure. To the authors’ knowledge, this is the first time that a method for this purpose has been fully described and characterised. Finally, to demonstrate this methodology, we have cultured two different cell types (HCT-116 colon carcinoma cell line and U251-MG glioblastoma cell line) in a hypoxic and nutrient-depleted microenvironment. We then recovered them at different time points for downstream characterisation of TME biomarkers and cell death mechanisms overtime via qPCR and AMNIS image cytometry in our microfluidic model.

## Results and Discussion

### Microdevice operation and workflow

**Figure 1:**
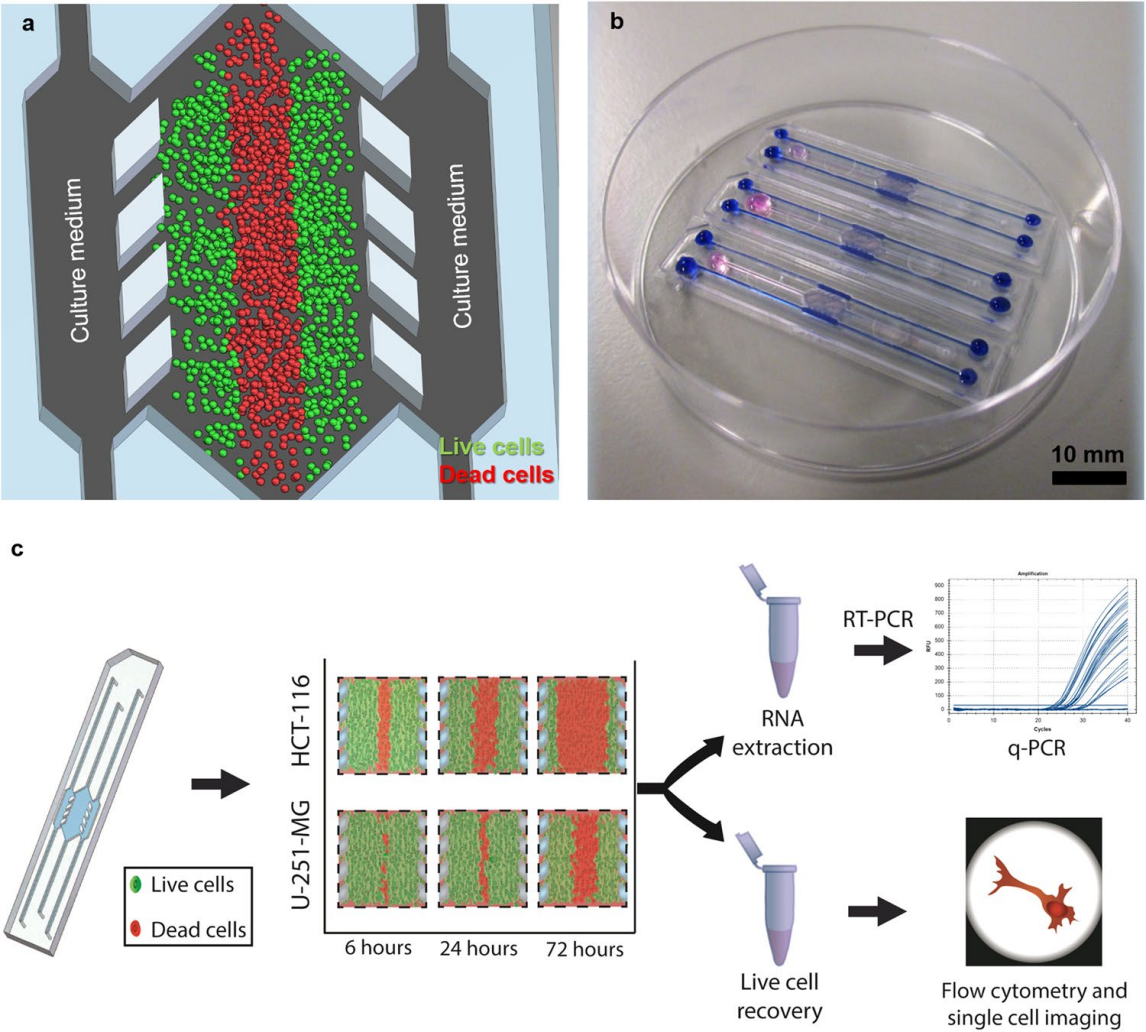
Experimental setup and description of the experimental procedure. (**a**) Schematic of the cell culture setup in a microfluidic device. Culture medium perfused through the lateral microchannels is the source of nutrients and oxygen and creates physiological gradients across the microdevice. Cells near the ‘surrogate’ blood vessels are viable, whereas oxygen/nutrient-depleted cells in the centre of the microdevice die, creating a ‘dead core’ similar to the necrotic regions found in tumours. (**b**) Photograph of the microdevices filled with hydrogel in the central chamber and blue/pink dyes are shown for visualisation purposes. (**c**) Schematic of the experimental procedure: After cultured within the microdevice for a specific amount of time, cells are extracted from the central microchamber via enzymatic degradation of the hydrogel and collected in an Eppendorf tube for subsequent RT-PCR and AMNIS image cytometry to characterise the expression different genes or proteins related to tumour metabolism.

For this paper, we used cyclic olefin polymer (COP) microfluidic devices designed with a central microchamber and two flanking lateral microchannels. The latter serve as surrogate blood vessels, and a series of posts delimit the connection to the central microchamber. This geometry, which has previously been described^23,34^, facilitates the confinement of a hydrogel for 3D cell culture within the central microchamber and the perfusion of culture media through the lateral microchannels without disturbing the confined hydrogel. COP a is transparent material that is impervious to oxygen diffusion, allowing for the creation and real-time monitoring of cell-generated oxygen and nutrient gradients in the central microchamber. Therefore, oxygen levels and nutrient availability are highest close to the lateral microchannels and decrease due to cell consumption as we move toward the centre of the microchamber (Fig. 1a,b). Overall, in this paper, we validated the extraction procedure for the further characterisation of this microfluidic model via qPCR and AMNIS image cytometry, as depicted in the workflow in Fig. 1c.

### Characterisation of hydrogel degradation kinetics

**Figure 2:**
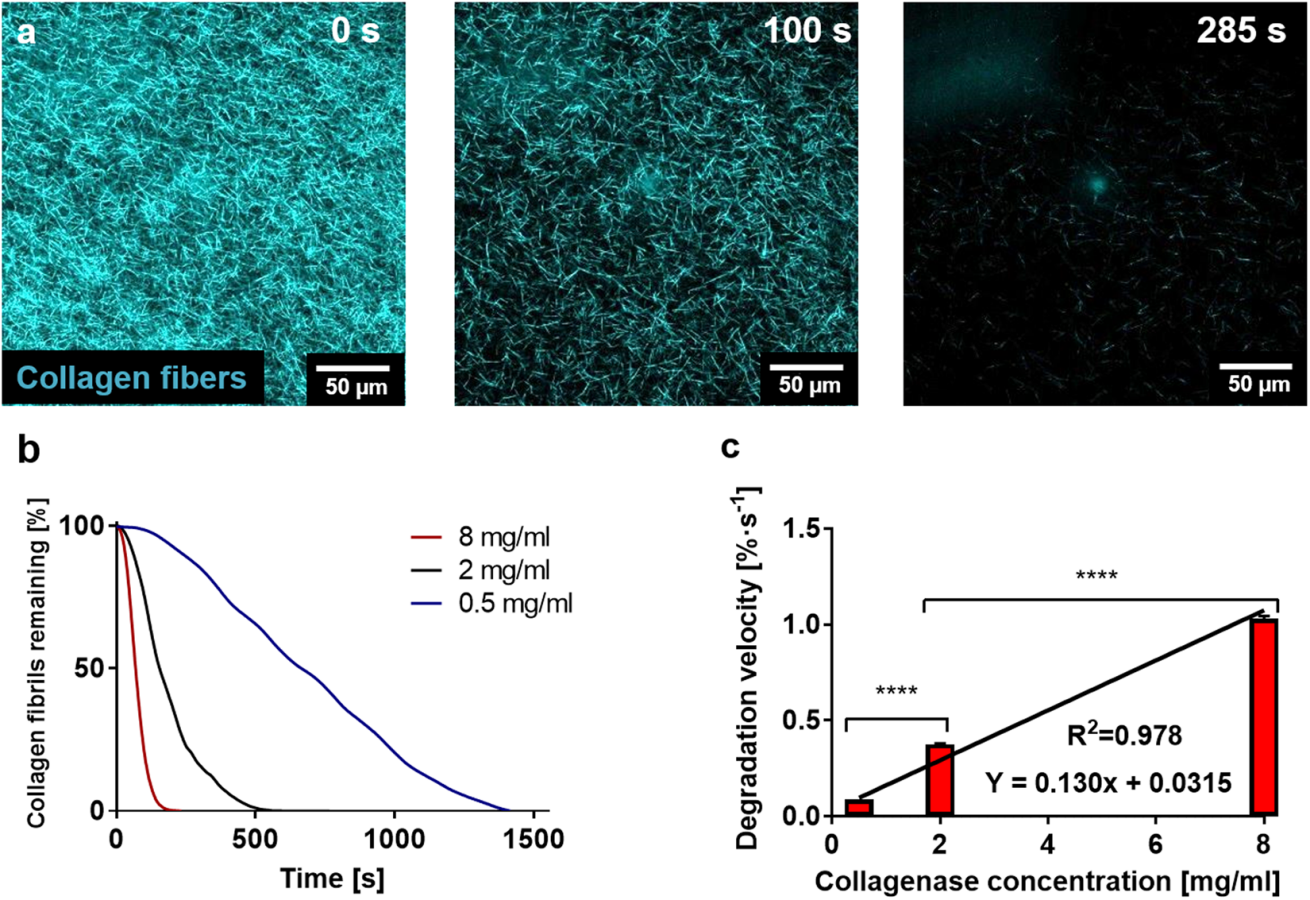
Optimisation of the enzymatic degradation of collagen hydrogels. (**a**) Confocal reflection microscopy of the degradation dynamics of a collagen hydrogel on a glass-bottomed Petri dish using an 8 mg/ml solution (18 IU/ml of collagenase P in PBS. The pictures show how the number of collagen fibrils constituting the hydrogel decreases over time. (**b**) Quantification of the hydrogel degradation dynamics for different collagenase concentrations through area quantification for 0.5, 2 and 8 mg/ml. (**c**) Characterisation of the degradation speed for the different collagenase concentrations. Linear fitting of the degradation speeds resulted in a linear equation (Y = 0.130x + 0.0315) R^2^ = 0.978. Graph shows average ± SEM (p-values < 0.001 in both cases).

To establish the cell recovery method, we first characterised the enzymatic degradation kinetics to optimise the method for microfluidic applications. We tracked the enzymatic degradation reaction utilising confocal reflection microscopy. After perfusing a collagenase solution through the lateral microchannels, the number of collagen fibrils progressively diminished over time until no fibrils were observed (Fig. 2a). We quantified, in real time, the area occupied by the collagen fibrils employing Fiji software and observed an exponential decay of the collagen fibre density (Fig. 2b). From this graph, we correlated faster degradation dynamics with higher concentrations of collagenase.

Furthermore, we fitted the linear fraction of the curve to a linear model and thus extracted the degradation velocities for each reaction. We found that the resulting graph was linear (Y = 0.130x + 0.0315) (Fig. 2c) and the constant term in this regression line is minimal, which strongly suggested that the concentration of collagenase is the only factor contributing to the velocity of degradation of the collagen hydrogel. All fittings performed in these experiments are detailed in Supplementary Table S1. Additionally, the degradation of the hydrogel can be better observed in the Supplementary Video S1.

### Efficiency of particle recovery from a confined hydrogel in a microfluidic device

**Figure 3:**
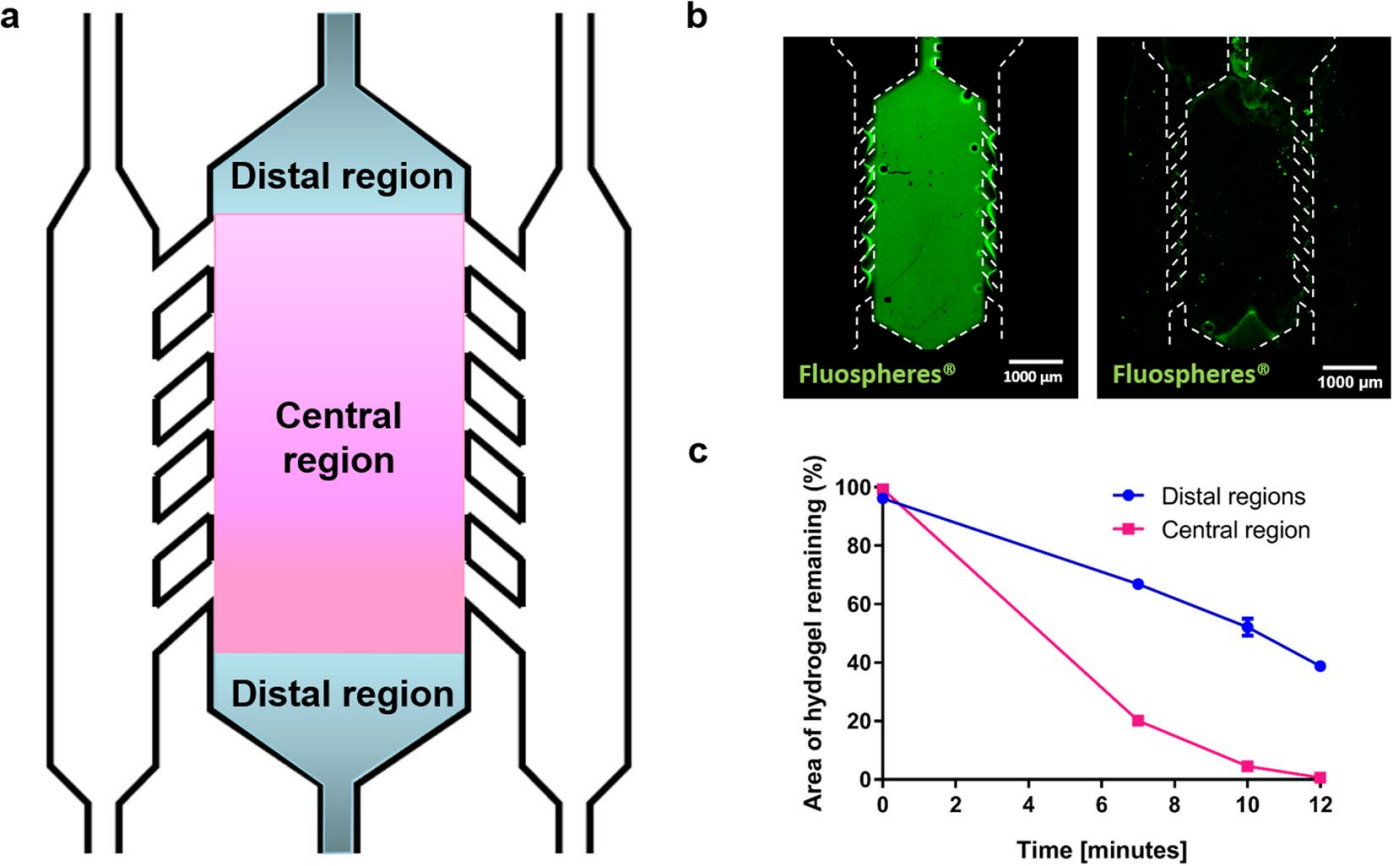
Recovery efficiency from the microdevices. (**a**) The microdevice was divided into three different regions for the analysis: the triangular regions on both ends of the central microchamber and the rectangular region flanked by the pillars. (**b**) Green-fluorescent FluoSpheres were embedded in a collagen hydrogel and confined in the central chamber of the microdevice. An 8 mg/ml collagenase solution was pipetted through the lateral microchannels and incubated at room temperature for 12 minutes. Images show the FluoSpheres before pipetting the collagenase solution and 12 minutes after addition of collagenase solution. (**c**) The area with remaining FluoSpheres was quantified for the central and distal regions and compared. Differences between distal and central regions were statistically significant at every time point excluding t = 0 (n = 3 and p < 0.0001, as found via two-way ANOVA and post-hoc Bonferroni tests).

After evaluating the hydrogel degradation, we chose to work with the highest collagenase concentration due to the high degradation velocity. However, the collagen hydrogel in a Petri dish is immersed in collagenase, making degradation occur at all the hydrogel interfaces, and therefore more quickly. In contrast, in the microfluidic device, hydrogel exposure to the collagenase solution occurs only from the lateral microchannels, making the process longer. Hence, we applied the chosen degradation conditions to a hydrogel confined within a microfluidic device, to validate that the collagen hydrogel could be fully degraded and particles within it could be retrieved in a practical timescale for a cell culture experiment. To test this, we observed the degradation of a collagen hydrogel confined within the central microchamber of the microdevice by imaging Green-fluorescent FluoSpheres embedded in the collagen hydrogel confined in the central microchamber using confocal microscopy. In this experiment, the release of the particles was correlated with hydrogel degradation. For quantification purposes, we divided the central chamber of the microdevice in 3 regions: the two triangular distal regions, located further from the microchannels (sources of nutrients and oxygen); and a central rectangular region, with more access to nutrients and oxygen (denoted “Distal regions” and “Central region” in Fig. 3a). After pipetting collagenase solution (8 mg/ml) we observed that the FluoSpheres were released from the gel in a timescale consistent with the described kinetics (Fig. 3b).

Furthermore, after pipetting the degraded hydrogel out of the microdevice through the lateral microchannels, we observed that the majority of the FluoSpheres were extracted from the central microchamber in a period of *ca*. 10 minutes. Only a fraction of those lying in the distal regions was not extracted as compared to the central region which, after area quantification, yielded a statistically significant difference (p < 0.0001) (Fig. 3c). Particularly for this design, nutrient and oxygen gradients are established within the central region, making the distal regions subjected to different cell culture conditions as compared with the central area. Therefore, selective extraction of the central area was desired to our subsequent assays. These results have been replicated with three other microdevice architectures (Supplementary results, Figs S5–S7). In all cases, particle recovery was over 80%.

### Effect of collagenase on cell viability

**Figure 4:**
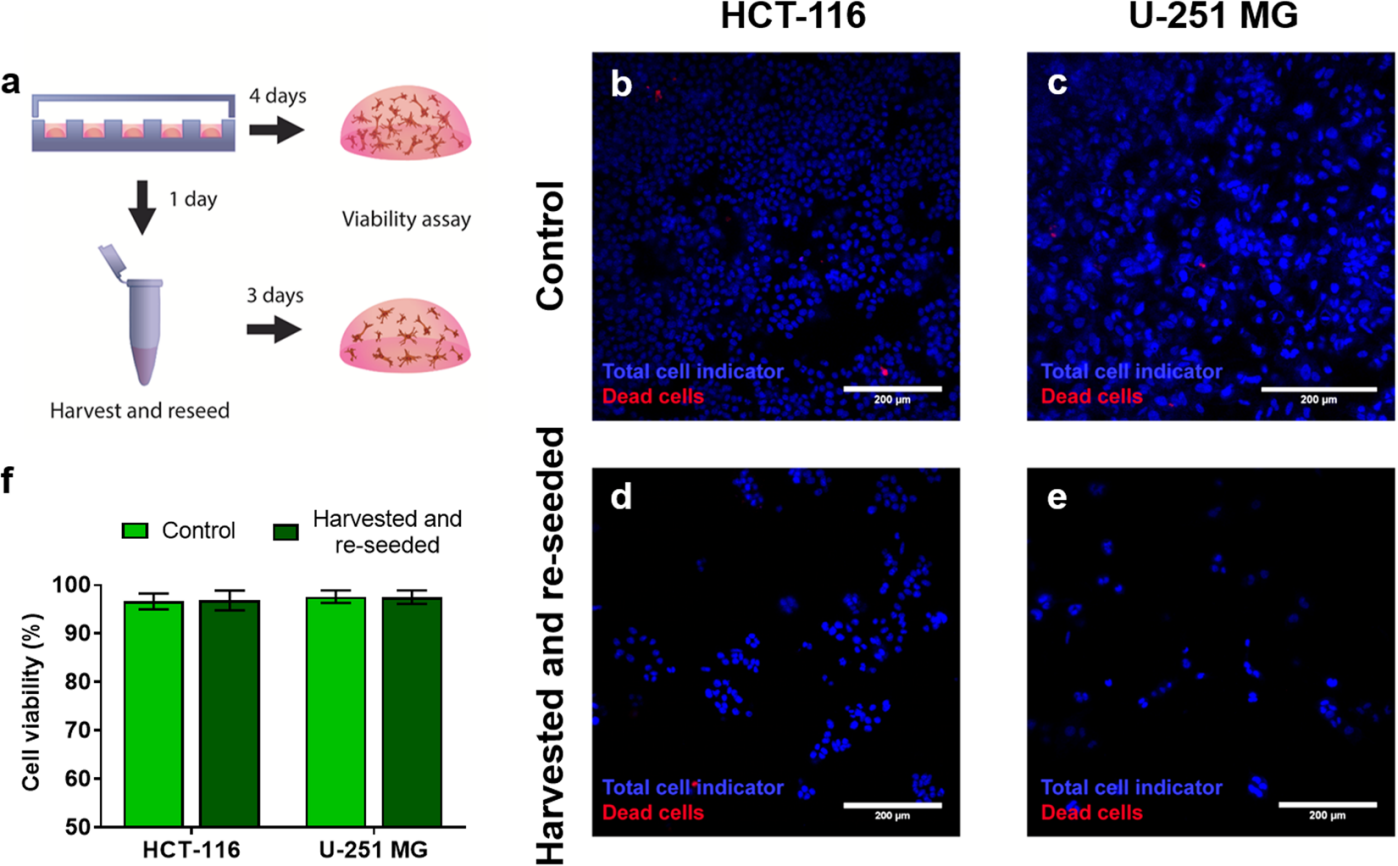
Biocompatibility of the cell extraction method. (**a**) Scheme of the experimental design. First, we embedded two different cell types independently in collagen hydrogels (HCT-116 and U-251 MG cells). After 24 hours, cells were extracted from the hydrogels by enzymatic degradation with an 8 mg/ml solution of collagenase in PBS. Recovered cells were washed with PBS and re-embedded in collagen hydrogels. Cell viability was assessed after 72 hours using confocal imaging, the cell nuclei were stained (Hoechst 33342, blue) and a dead cell indicator was added to distinguish dead cells (propidium iodide, red). Results from extracted reseeded cells were compared with non-treated cells (control). Images of the control cells can be observed in (**b**) for HCT-116 cells and (**c**) for U-251 MG cells. Images of the recovered and re-embedded cells can be observed in (**d**) for HCT-116 cells and (**e**) for U-251 MG cells. (**f**) Quantification of cell viability for control and extracted and re-embedded cells, for both mentioned cell types used in this study (P-value > 0.67 for HCT-116 cells and p-value > 0.99 for U-251-MG cells as determined via two-way ANOVA and post-hoc Bonferroni test).

After establishing the degradation kinetics and method, we proceeded to examine the effect of the collagenase on cell viability. We used two distinctive cell types and embedded them in a collagen hydrogel. Next, we extracted the cells using the highest collagenase concentration used during the degradation experiments (8 mg/ml) and re-embedded them again in a collagen hydrogel (Fig. 4a). We evaluated cell viability by confocal microscopy with a viability stain after 72 hours and compared the re-embedded cells to control conditions (i.e. cells never extracted from the hydrogel) (Fig. 4b–e). No significant impact was observed on cell viability for any of the cell types used in this study (Fig. 4f). A lower cell concentration is shown in the harvested and reseeded samples. This decrease was expected since the cell suspension recovered from the hydrogels was diluted in the re-seeding process. The cells in the control hydrogels did not experience this dilution, since they remained the same throughout the experiment, resulting in a higher cell concentration for the control hydrogels than for the “harvested and reseeded” ones.

To avoid bias in our experiment that could incur in the discrimination of dead cells due to washing, we performed an additional set of experiments that avoids these steps. We performed fluorescence-based time-lapse imaging both the degradation of a 3d droplet-format hydrogel and an untreated control with cell tracker CMFDA and PI (Video S1). Therefore, in the images all cells are stained in green, whereas cells with membrane damage fluoresce in red. First, we quantified cell viability overtime at 0, 4, 8, and 12 min in this time lapse (Fig. S2c). Cell viabilities ranged from 98.3 ± 0.332% (at time 0 min) to 98.9 ± 0.245%. The viability decrease was 0.6% between these two time points and was found not significant.

After 20 min, we imaged with confocal microscopy both the collagenase-treated suspension and the control hydrogel, without removing the collagenase solution (Fig. S2a,b). We found no significant differences in viability after 20 min between those conditions (98.9 ± 0.309% for the collagenase-treated hydrogel and 98.2 ± 0.313% for the control). This 0.7% decrease indicates that cells do not suffer any significant change in viability due to collagenase incubation in 20 min in our setup.

While cell viability was not affected by the procedure, it could incur in a reactive oxygen species or apoptosis response in recovered cells. Hence, we investigated the impact of the collagenase degradation on cell stress and rates of apoptosis (Supplementary Fig. S1a,b). For the assessment of early apoptosis levels, we compared the results obtained from control hydrogels (i.e. never exposed to collagenase) and from harvested and reseeded hydrogels (Supplementary Fig. S1c), and we found no statistically significant differences between both conditions (3.789 ± 0.6363% and 3.395 ± 0.4637% respectively, p = 0.6265, Samples passed Shapiro-Wilk normality test and were subjected to Student’s t test with Welch correction). As for the assessment of oxidative stress levels, the quantification relies on fluorescence intensity, which is an arbitrary measurement. Therefore, we calculated the ratio fluorescence intensity in harvested and reseeded over fluorescence intensity in control samples (Supplementary Fig. S1d). The results yielded a ratio of 1.32 ± 0.127, not significantly different from the reference value (in this case, 1). Likewise, we compared these results with an internal control of cells in 2D treated with 3% or 5% of DMSO for 4 hours, since DMSO has been reported in the literature to induce ROS production^36^. The results, very far from the reported increase in our experiment, were 2.198 ± 0.3978 and 3.877 ± 0.4334. These results were significantly different from the reference value (p = 0.0506 and p < 0.0001, respectively), as assessed via one-way ANOVA and Tukey post-hoc test.

Overall, we decided to continue using the highest collagenase concentration (8 mg/ml) for cell recovery from the microdevices, since it significantly accelerates the process and shows no damage to cell viability or cell stress induction after 72 hours.

### Microfluidic model of the TME

**Figure 5:**
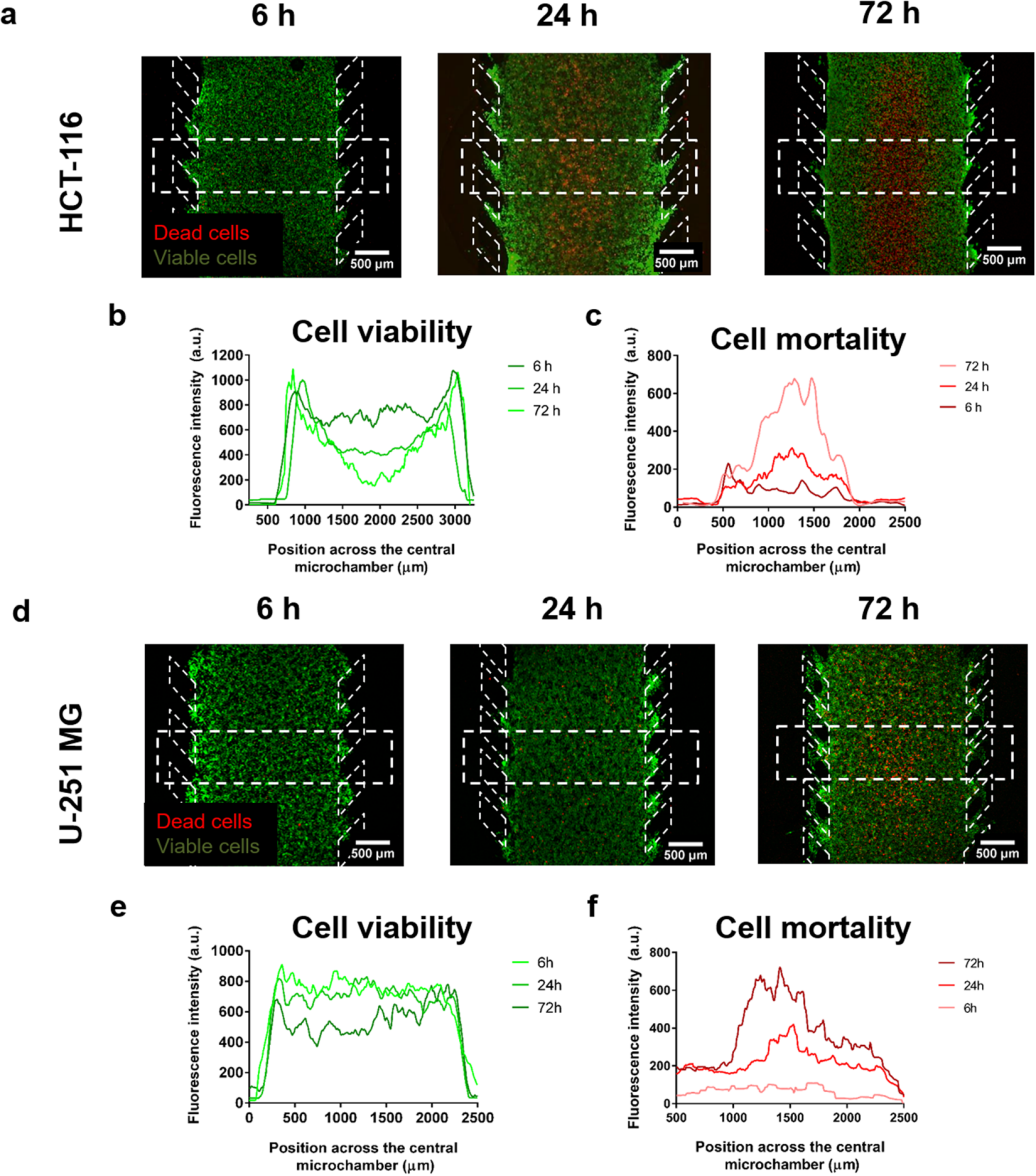
Dead cell core generation within the microdevice. Cells were embedded within the collagen hydrogel at a final concentration of 40 million cells/ml in the central microchamber. Cell viability was evaluated for HCT-116 cells (**a**) and U-251-MG cells (**d**) at different times (6 h, 24 h and 72 h) using propidium iodide (PI) and Calcein-AM (CAM) staining. Viable cells are depicted in green, whereas dead cells are depicted in red. Furthermore, cell viability profiles (**b**,**e**) and cell mortality profiles (**c**,**f**) were obtained for HCT-116 and U-251-MG cells from the rectangular region of interest shown in the corresponding images.

HCT-116 colon cancer cells were embedded in a collagen hydrogel in the central microchamber of the microdevice at 4·10^7^ cells/ml, and cell viability was evaluated at different time points (6, 24 and 72 h). As previously described, we observed the appearance of a core of propidium iodide (PI) positive cells, cells with loss of cell membrane integrity, flanked by viable cells (i.e. calcein-AM (CAM) stained regions) (Fig. 5a). This region of PI-positive cells appeared after 24 hours of culture and grew thicker after 72 hours in culture. Images were quantified via plot profiles to observe the changes in PI and CAM signal in the cell cultures over time (Fig. 5b,c).

When this experiment was duplicated with U-251 MG cells (Fig. 5d), we only observed the appearance of a dead core after 72 hours of culture (Fig. 5e,f); illustrating the differences of both cell types regarding nutrient and oxygen consumption.

### Analysis of extracted cells with downstream RNA extraction and qPCR

Finally, we validated the cell recovery protocol by subjecting recovered HCT-116 cells to two different downstream analysis techniques: qPCR and AMNIS image cytometry.

**Figure 6:**
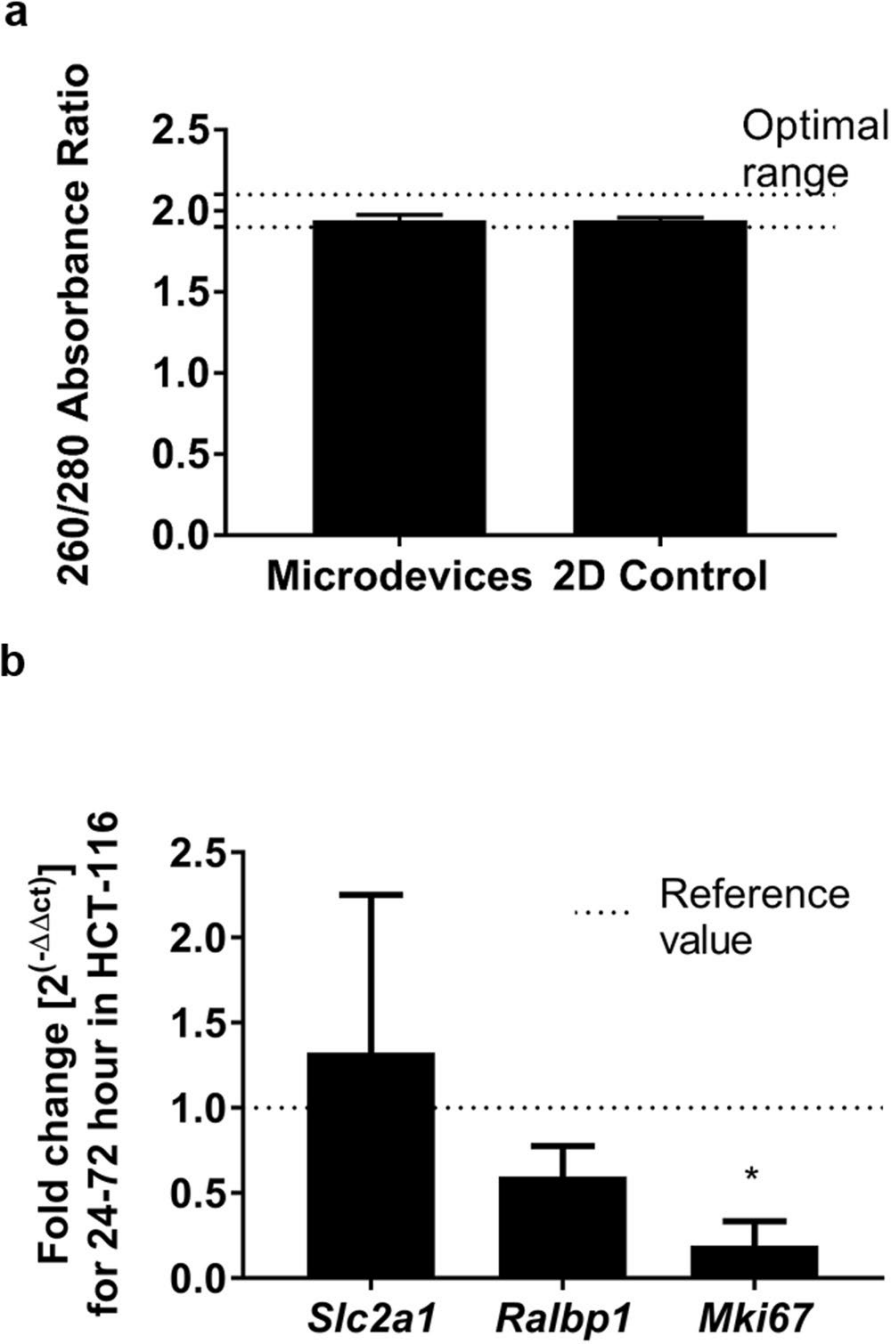
Validation of the degradation method for downstream qPCR. (**a**) 260/280 absorption ratio was determined for RNA extracted from cells seeded in 2D (2D control) and from cells seeded in microdevices. No significant differences were found (Samples passed Shapiro-Wilk normality test, and Student’s t-test was performed) (p = 0.8604). (**b**) qPCR validation of extracted RNA. Expression fold changes in *Slc2a1* (Glut-1), *Ralbp1* (ralA binding protein 1) and *Mki67* (Ki-67) were assessed between 24 and 72 h in samples recovered from the microdevice with the described method. *Actb* and *Gapdh* were used as reference (housekeeping) genes for normalisation. Only *Mki67* yielded significantly different results from the reference value (Samples passed Shapiro-Wilk normality test and were subjected to two-way ANOVA with FDR method of Benjamini, Krieger and Yekutieli) (p = 0.012).

First, we extracted RNA from cells recovered from microdevices and 2D control samples. To check for possible protein quantification that could affect RNA quality and eventually qPCR, we performed 260/280 absorbance ratio measurements for these two different conditions (Fig. 6a). Pure RNA produces 260/280 ratios around a value of 2, and lower values are indicative of high protein content in the sample. The results of this experiment revealed that the average 260/280 ratio was 1.943 ± 0.07353 (average ± SEM) for control cells lysed from a 2D Petri dish, whereas, for cells recovered from the microdevices, the average ratio was 1.946 ± 0.01323. Differences were found to be non-significant between both conditions, indicating that the RNA extraction kit is sufficient to eliminate the protein excess present in the digested samples, as a consequence of the collagenase solution and collagen traces.

Next, we performed qPCR on the extracted samples, and calculated fold changes between 24 and 72 hours within the microdevice (Fig. 6b). The 6-hour samples were disregarded to allow cells to adjust their gene expression to the new 3D environment. First, we studied cell proliferation via quantification of *Mki67* expression (Ki-67). *Mki67* expression variation was 0.191 ± 0.072, meaning that the expression decayed at 72 h as compared to at 24 h. This decrease was statistically significant (p = 0.012) indicating that cell proliferation greatly diminished as time passed in cell culture, which is consistent with previously reported evidence^31^ that showed impaired cell proliferation in the microdevice chamber using real-time fluorescent reporters, except for the regions closest to the lateral microchannels (i.e. the sources of nutrients and oxygen). Next, we assessed the changes in expression of *Slc2a1* (Glut-1, an indicator of glucose uptake^37^). We found that the expression of *Slc2a1* suffered an increase of ~30% (1.324 ± 0.535), that was found non-significant (p = 0.775). Then, we examined expression changes in *Ralbp1* (ralA binding protein 1, which is a stress response protein and stress protector^38^). The expression fold-change of *Ralbp1* was 0.596 ± 0.090, which yielded a non-significant difference (p = 0.393)). These results did not demonstrate changes in glucose uptake and stress responses in the TME model between 24 and 72 hours. Overall, these results do not suggest the implication of these genes in the cell responses shown by our model.

As an internal control, we compared microdevice gene expression data with 2D controls (Supplementary Fig. S2). Results revealed that *Slc2a1* produced a similar fold change in 2D between 24 and 72 hours (1.324 ± 0.535, p = 0.3023), that yielded non-significant results when compared to microdevice fold changes. In the case of *Mki67*, the 2D control was not significantly different from the reference value (0.751 ± 0.067, p = 0.9114) but was borderline significantly different from the microdevices (p = 0.075). These results are consistent with literature data, indicating that proliferation is decreased in 3D systems as compared to 2D cell cultures^39^. Conversely, *Ralbp1* suffered a dramatic increase in expression, significantly higher than its microdevice counterpart (1.952 ± 0.210, p<0.0001). This could indicate lower microenvironmental stress since nutrient and oxygen gradients are trivial in the 2D group. Overall, these data are consistent with the literature, that has provided ample evidence of gene expression differences between 2D and 3D culture systems^40–43^, as well as the starvation conditions, that have shown to impact cell behaviour and gene expression deeply^44^.

### Analysis of extracted cells with AMNIS image cytometry

**Figure 7:**
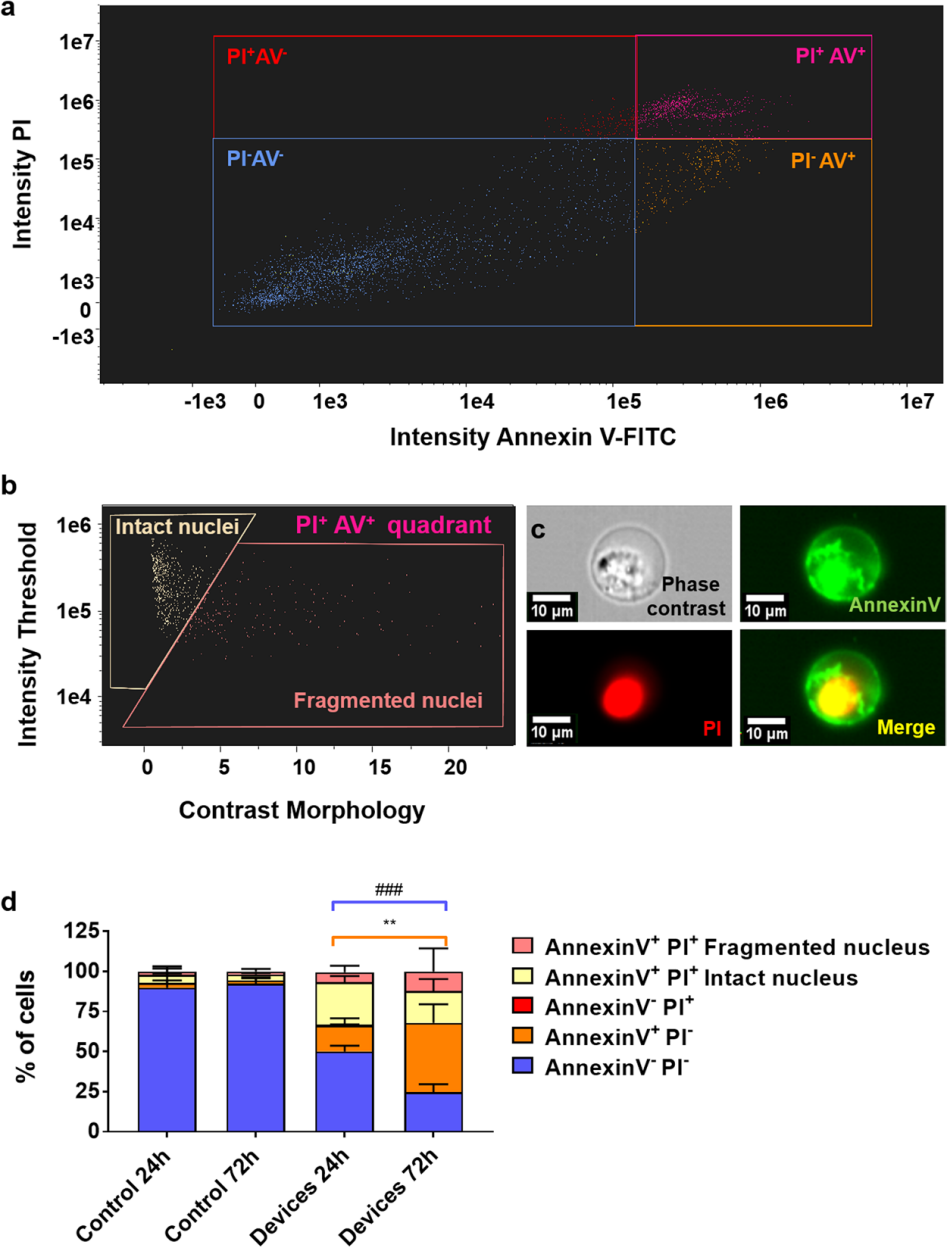
Validation of the degradation method for downstream AMNIS flow cytometry with individual cell imaging capabilities. (**a**) Sample flow cytometry 2D plot of the cell death analysis from cells extracted from a microfluidic device after 72 h of culture. (**b**) Breakdown of Annexin V^+^PI^+^ cells from the plot shown in 7a and elucidation of their phenotype according to contrast morphology classification of nucleus integrity. (**c**) Single-cell sample fluorescent images of a recovered necroptotic cell (Annexin V^+^PI^+^ and solid nucleus in PI staining channel). Phase contrast (top-left), Annexin V-FITC (top-right), Propidium iodide (bottom-left) and merge of the two latter (bottom-right). Single-cell images were used for discerning cell phenotypes, including necroptotic (intact nucleus) and apoptotic (inhomogeneous nucleus) phenotypes among Annexin V^+^PI^+^ cells. (**d**) Graph showing the measured percentage of different cell phenotypes in the microdevices after 24 and 72 hours, according to Annexin V and PI staining and nucleus integrity for Annexin V^+^PI^+^. Controls in 2D at 24 and 72 hours are also shown to validate the staining and cell viability. Annexin V^−^PI^−^ and Annexin V^+^PI^−^ yielded statistically significant results after a two-way ANOVA (**p-value = 0.0011 and ^###^p-value = 0.0006 respectively) with a Tukey correction for multiple comparisons. Conversely, the controls were not significantly different from each other (p-value > 0.96 in all cases) and demonstrated the staining for all the described populations.

Finally, we performed AMNIS image cytometry on recovered HCT-116 cells from microdevices at 24 and 72 h after cell seeding and compared the results. This technique captures single-cell images simultaneously to performing traditional flow cytometry. We performed controls in 2D to validate the staining and demonstrate cell viability of the cells in the absence of oxygen and nutrient restrictions. Figure 7a shows a 2D plot of cells extracted from a microfluidic device after 72 hours of culture (examples of staining can be seen in Supplementary Fig. S3). The 2D plot presents the results for double staining of Annexin V (AV) and propidium iodide (PI), yielding the following populations: AV^−^PI^−^ (consistent with live cells), AV^+^PI^−^ (consistent with early apoptotic cells), AV^−^PI^+^ (consistent with necrotic cells). Regarding the double positive population (PI^+^AV^+^, top right of Fig. 7a), this population corresponds with both necroptosis and late apoptosis^45,46^. Hence, a contrast morphology of the nucleus was performed to separate cells with an intact nucleus (necroptotic cells) from cells with a ruptured or inhomogeneous PI staining of the nucleus (late apoptotic cells). A 2D plot with a breakdown of the PI^+^AV^+^ quadrant can be observed in Fig. 7b, whereas a sample of the single cell images with all the populations defined by their staining can be found in Fig. 7c. Finally, the overall results for the different populations can be observed in Fig. 7d, as well as 2D controls at 24 and 72 h to demonstrate cell staining and plain cell viability of cells when not subjected to starvation or oxygen deprivation.

Firstly, the controls in 2D showed no significant difference in any of the populations, indicating that cell viability remained very high (89.8 ± 9.05% and 92.6 ± 3.89% respectively for AV^−^PI^−^, that correlates with live cells) in the absence of starvation or oxygen deprivation over time. Likewise, we observed both positives and negatives for AV and PI in controls, indicating that the staining was successful for both markers.

Next, we compared populations yielded by the double staining in the microdevice samples at 24 and 72 hours of culture. We found significant differences for the live cell population (AV^−^PI^−^), decreasing a 51.6% in the 72-hour sample (24.7 ± 4.95%) as compared to the 24-hour sample (50.0 ± 3.68%). This decrease is consistent with the higher mortality shown in Fig. 5a,b.

After that, we compared the cell death populations between the 24 and the 72-hour sample, finding that the necrosis population (AV^−^PI^+^) remained negligible in both conditions. As for the AV^+^PI^−^ populations, neither the necroptotic population (intact nucleus) nor the late apoptotic population (inhomogeneous nuclear staining) changed significantly between the assessed samples. However, the slight increases experimented by these populations may account for the increase in the PI^+^ cell population shown in Fig. 5a,b.

Interestingly, the early apoptosis population (AV^+^PI^−^) increased 2.6-fold from the 24-hour sample to the 72-hour sample (16.4 ± 4.31% and 43.15 ± 11.7%), increasing cell death in a manner not detectable by the assay presented in Fig. 5, which relied only on PI as a marker. This cell death increase is also consistent with the sustained low oxygen and deprivation. Overall, these results indicate that cell viability decreases between 24 and 72 hours of culture in favour of early apoptosis. These findings are consistent with prior evidence from other groups^47^, that indicate the effect of starvation of the tumour microenvironment could promote apoptosis in cancer cells regardless of their p53 status. In this context, the workflow presented in this paper would be a good candidate for *in vitro* testing of apoptosis-inducing drugs in conditions more similar to the tumour microenvironment^47–49^.

## Conclusions

In this paper, we have developed, characterised for the first time a simple and user-friendly enzymatic protocol that allows for the recovery of embedded cells from a microfluidic device in *ca*. 10 minutes. The described protocol is efficient and does not affect cell viability or generate significant amounts of stress on recovered cells. Additionally, the incubation time of collagenase has been optimised for our particular model, providing a robust protocol for cell extraction and downstream analysis.

Importantly, we have demonstrated that, as well as maintaining cell viability, cells can be used for downstream applications, such as qPCR and flow cytometry, hence making it possible to perform additional techniques on embedded cells after their release from a microfluidic device and expanding the possibilities of microfluidic studies performed in rigid and oxygen impervious devices.

Furthermore, we have used a microfluidic model in which, by modifying cell culture time, we can tune the size of the region occupied by necrotic cells. Cell behaviour, gene expression and cell fate are modified in this setup in a self-standing and continuously evolving microenvironment, making it an ideal platform to validate the cell recovery protocol. Next, the described protocol made it possible to couple microfluidics with AMNIS flow cytometry and single cell imaging, allowing the collection of data on single cell morphology, cell surface marker expression and in this case, cell death profiling, which could also be of interest for drug testing applications and fundamental studies of the TME influence in cancer. The optimization of this protocol enabled us to identify several differences in gene expression as compared to 2D controls, such as the marked decrease in Ralpb1 in the microdevices as compared with the 2D. Likewise, we observed how the expression of *Mki67* plummeted between 24 and 72 hours of culture in the microdevices. This observation is consistent with previous literature from our group, indicating that proliferation decreased in the majority of the chamber of the microfluidic model^31^. Finally, we have identified a significant increase in death via early apoptosis in the period between 24 and 72 hours of culture in the microdevices, which would contribute to the increased mortality shown in microdevices in our model.

Overall, these results add up to the development of microfluidics for cell-based studies. We expect that this method can be adapted to many different platforms and set-ups and help bridge the gap between traditional molecular biology and microfluidics, enabling the study of tumour cell behaviour in more realistic models of the tumour microenvironment, hence resulting in more efficient therapies. eventually enabling the promise of using the TME to battle cancer.

## Materials and Methods

### Microdevice fabrication and handling

Cyclic olefin polymer-based microdevices were produced by mould injection and bound to a petri dish with biocompatible adhesive (Adhesive Research). The design comprised a 2000 μm-wide central chamber, flanked by two 700 μm-wide, 250 μm-deep lateral microchambers. The central microchamber was delimited from the lateral microchannels by parallelogram-shaped pillars that allowed liquid and cell confinement within hydrogels as described elsewhere^22^. Hence, lateral microchannels remained hydrogel-free and were filled with media or collagenase solution when needed. Hydrogel injection was performed through dedicated inlets and outlets by manual pipetting. The configuration of the microdevice is shown in Fig. 1a.

Microdevices were fabricated by injection moulding and attached to Petri dishes using biocompatible adhesive. Before their use in cell culture, microdevices were sterilised with 70% ethanol. After air-drying and 2 h UV exposure, microdevices were ready for use.

A ‘3D cell culture’ system with injected hydrogel was confined to the central chamber. The lateral microchannels remained hydrogel-free and effectively served as ‘surrogate blood vessels’, as well as an entry point for cell staining and collagenase solutions.

### Hydrogel preparation and setup handling

#### Hydrogel preparation procedure

Type I rat tail collagen hydrogels were prepared to embed the cells within the microdevices. Reagents were kept in ice to prevent premature gelation. Cells were trypsinised and resuspended to the desired cell concentration. Using a chilled tip, a mixture of 33.8 μl collagen type I (3.35 mg/ml, Corning 354236); 0.85 μl NaOH 1 M (Sigma 655104); 10 μl DMEM 5X (Sigma D5523), 50 μl culture media (preparation described in the Cell culture section) and 5.35 μl distilled sterile water was prepared.

#### Hydrogel droplet preparation

For viability assays we performed the experiments using in a hydrogel droplet format, a chilled tip was used to pipette one 3 µl droplet of cell suspension in collagen mixture described in the previous section per well in a 96 well plate (Sarstedt, 82.1581.100). At least 4 droplets were prepared for each condition and assay. The plate was then placed into an incubator (37 °C and 5% CO_2_) for 15 minutes to allow for collagen polymerisation.

#### Hydrogel preparation in the microdevice

The hydrogel mixture, prepared as described above, was injected into the microfluidic device employing a micropipette. Additionally, 10 μl of the hydrogel mixture droplet was placed on top of the inlet to prevent hydrogel evaporation. The filled microfluidic device was subsequently placed into an incubator (37 °C and 5% CO_2_) for 15 minutes to allow for collagen polymerisation. 5 ml of culture medium was added to cover all the microdevices attached to the Petri dish. Culture medium was perfused through the lateral microchannels to allow oxygen and nutrient diffusion. In all experiments, the culture medium in the lateral microchannels was refreshed once a day by pipetting 10 µl through each lateral microchannel.

When a different culture medium or solution (e.g. viability staining or collagenase solution) was desired, the culture medium was removed from the Petri dish by aspiration, 5 ml of the new medium was added, and 10 µl were manually pipetted through the lateral microchannels.

### Maintenance cell culture

Human cell lines HCT-116 (colon carcinoma) and U-251 MG (glioblastoma) were purchased from the American Type Culture Collection (ATCC) and routinely cultured in high glucose Dulbecco’s Modified Eagle’s Medium (DMEM) (Lonza, BE12-614F) supplemented with 10% Fetal Bovine Serum (FBS, Sigma-Aldrich F7524, non-USA origin), 2 mM L-glutamine (Lonza, 17–605 C) and penicillin/streptomycin (100 units/ml and 100 µg/ml respectively) (Lonza, DE 17–602E) within a TEB-1000 humidified 5% CO_2_ incubator (EBERS Medical Technology) at 37 °C.

### 3D embedded cell culture

Hydrogel mixtures were prepared as described in the previous section to embed cells in the hydrogel, except for the cell culture media, which was replaced by cell suspension. Hence, cells were routinely trypsinised and resuspended at a concentration of 80 million cells/ml, to yield a final concentration of 40 million cells/ml. The cell suspension was added to the hydrogel mix and thoroughly mixed by pipetting up and down before incorporating to the desired setup.

### Hydrogel degradation procedure and cell recovery

Collagenase P (Roche,11213857001, 2.3 IU/mg) was dissolved in PBS to a final concentration of 8 mg/ml (18 IU). Collagenase solutions were subsequently sterile-filtered and added to the hydrogels. For degradation of hydrogels in well plates, 100 μl were added per well. For degradation of hydrogels within microdevices, 50 μl of the collagenase solution were pipetted through each lateral channel of the microdevice as described in previous sections. Collagenase incubation was performed at room temperature for 10 min. Afterwards, the recovered cell suspension was transferred to a fresh Eppendorf tube and washed with PBS before subsequent steps.

### Hydrogel degradation tracking

5 µl collagen hydrogels at 1.2 mg/ml were prepared in a glass-bottom Petri Dish (Ibidi, 81158) as described above, and let stabilise overnight in an incubator. Confocal reflection images were taken using a Nikon Eclipse Ti microscope equipped with a C1 modular confocal microscope system. The barrier filter of a 450/35 nm filter cube was removed to enable reflection mode, and the sample was visualised using a 488 nm laser. In this configuration, hydrogel reflected laser light was captured by the photomultiplier tube, hence allowing the visualisation of the collagen fibres. Collagenase P (Roche, 11213857001, 2.3 IU) was dissolved in PBS to final concentrations of 0.5, 2, and 8 mg/ml. Collagenase was added to the gels and incubated at room temperature for 5–25 minutes. The degradation process was tracked during this time, acquiring images as fast as possible in the system.

For the additional geometries, we repeated this procedure using two concentrations of collagen I: 2 and 6 mg/ml. We chose to use these concentrations because they are widely used in *in vitro* cell culture studies. Likewise, lower concentrations did not perform well in all devices. To track this procedure, we added FluoSpheres to the hydrogel mixture (as described for Fig. 4) and 10% of the collagen was substituted for fluorescently-conjugated collagen (kindly provided by Dr Brian Burkel). 8 mg/ml collagenase was added to the gels and incubated at room temperature for 5–25 minutes.

Image analysis was performed with Fiji (http://fiji.sc/Fiji). Time-lapse images were binarised, and the fibril occupied area was quantified over time and plotted for each collagenase concentration.

### Cell viability and stress quantification

To assess cell viability of the degradation procedure, individual cells need to be distinguished. Thus, we used bisBenzimide H 33342 trihydrochloride (Hoechst33342, Sigma-Aldrich B2261) and propidium iodide (PI, Sigma-Aldrich P4170) for cell staining. A Hoechst33342 stock solution was prepared by dissolving in DMSO to a concentration of 10 mg/ml, whereas PI was dissolved in distilled water to a final concentration of 2 mg/ml. Stock solutions were diluted 1:10000 and 1:1000 respectively in phosphate-buffered saline (PBS, Lonza BE17-516F) and gels were incubated with the solution for at least 15 minutes in the dark before imaging. Cells were visualised by confocal microscopy (Nikon Ti-E coupled to a C1 modular confocal microscope). All cells were stained blue (Hoechst33342) whereas only dead cells (PI-positive) were stained red. PI cannot penetrate living membranes, so it is only incorporated into cells with disrupted membranes.

To evaluate the incidence of cell stress responses due to the degradation procedure, we stained cells with CellROX Reagent (ThermoFisher, C10422), for the detection of oxidative stress, CellEvent Caspase-3/7 Detection Reagent (ThermoFisher, C10423) for the detection of early apoptosis according to the manufacturer’s instructions. Hoechst33342 was also used for nuclear staining. Positive controls for ROS increase were produced by supplementing control samples with DMSO 5% or 3% for 4 h, as described previously^36^.

### Cell viability for tumour development tracking

For tumour development tracking, single cell counting was not necessary. Hence, Hoescht33342 was substituted by a Calcein-AM solution. Stock solutions of 5 mg/ml Calcein-AM (CAM) (Life Technologies, C1430) and 2 mg/ml propidium iodide (PI) (Sigma P4170) were dissolved in DMSO and distilled water respectively. To test cell viability within microfluidic devices and in Petri dishes, stock solutions of CAM and PI were diluted to 5 and 4 μg/ml, respectively, in phosphate-buffered saline (PBS) (Lonza BE17-516F). CAM/PI solution was perfused through the lateral microchannels. Cells were then visualised by confocal microscopy (Nikon Ti-E coupled to a C1 modular confocal microscope), with viable cells (CAM-positive) stained in green and dead cells (PI-positive) stained in red. Cell viability profiles were evaluated by analysing the fluorescence intensity of the viable/dead cells across the central microchamber. All confocal images were taken at different focal planes with subsequent image analysis performed using ImageJ software.

### Gene expression assays

#### RNA extraction

Embedded cells were extracted from collagen hydrogels confined in 2 microdevices, as described in previous sections. 2D control samples were extracted from 12-well plates at 150,000 cells/well. RNA was extracted by using Total RNA Purification Plus Kit (Norgen Biotek Corp, Canada). The lysis step was performed to harvested cells according to the manufacturer’s instructions and the resulting suspension was stored at −80 °C for later RNA extraction. Once extracted as directed, RNA concentration and purity were assessed employing absorbance measurements using a Biotek Synergy HT multi-reader (BioTek Instruments, Inc. VT, USA).

#### cDNA

cDNA was synthesised by using PrimeScript RT-PCR Kit according to the manufacturer’s instructions. Briefly, 1 μg of RNA was taken from each sample and diluted to a final volume of 16 μl in an Eppendorf tube. 4 μl of 5x Master mix was added to a final volume of 20 μl. The mixture was incubated in a CFX96 Real-Time System (Bio-Rad, Spain) for 15 minutes at 37 °C, for 5 seconds at 85 °C and finally, the mix was cooled down to 4 °C.

#### qPCR

PrimeTime qPCR assay probes were purchased from IDT (Integrated DNA Technologies, Spain) for the genes presented in Table S2, where primers are also detailed. Primers were resuspended at a 10x concentration (10 µM), whereas probes were resuspended at a concentration of 5 µM, both in Nuclease-free water according to the manufacturer’s instructions and stored at −20 °C until use. Screened and reference (housekeeping) genes, along with their forward and reverse primers (in that order), are detailed in Supplementary Table S2.

PrimeScript qPCR Kit was used to perform the qPCR reactions in a CFX96 Real-Time System according to the manufacturer’s instructions to a final qPCR volume of 10 µl. The conditions for the q-PCR were the following: 95 °C for 15 s, [95 °C for 15 seconds, 60 °C for 30 seconds] (40 cycles), followed by a 4 °C cooling hold step. Two endogenous genes (*Gapdh* and *β-actin*) were used for normalisation of the data. All reactions were performed in triplicate, and all reaction efficiencies of the primer/probe sets were close to 100%. Target gene expression was normalised using the geometric mean of the mentioned reference genes and relative gene expression fold changes were determined between the time points of 24 and 72 h using the 2^−ΔΔCt^ method.

### AMNIS image cytometry

The determination of cell mortality and its classification between early, late apoptosis, necrosis and necroptosis was carried out in the Service of Citomics of the University of Zaragoza with Image Cytometer “ImageStream X” (AMNIS, Seattle, WA) as previously described in the literature for the determination of cell viability along with cell death studies^45,46^. Cells grown in the microdevices for 24 and 72 h were extracted from the devices with the collagenase technique described above. After one PBS wash and centrifugation, cells were resuspended in PBS and stained with “FITC Annexin V apoptosis detection Kit with Propidium Iodide” (Biolegend, 640914) according to the manufacturer’s instructions. The staining kit was validated with HCT-116 cultured in 2D for 24 and 72 h as an internal control.

For cell analysis, the 488 nm laser was used. The capture of the fluorescence of 480–560 nm was used for the determination of Annexin V (AV) and the capture of 660–745 nm for the determination of propidium iodide (PI). The representation of both contents determines in the four quadrants of the bi-parametric histogram: the number of living cells without staining (PI^−^AV^−^), early apoptosis (PI^−^AV^+^), dead due to necrosis or necrotic (PI^+^AV^−^). The population with double staining (PI^+^ and AV^+^) underwent an additional determination of nucleus morphology. For nucleus morphology, we used both the area occupied by the nucleus and bright detail intensity to discriminate necroptotic cells (intact nucleus with homogeneous staining) from late apoptotic cells (fragmented and inhomogeneous nucleus).

### Image analysis

Laser confocal and fluorescence images were acquired with a Nikon Eclipse Ti-E C1 confocal microscope. Images were analysed using Fiji software (http://fiji.sc/Fiji)^50^. Fluorescence intensity across the central microchamber of the microdevice was quantified in the different experiments by selecting a rectangular region across the central microchamber. The fluorescence intensity across that section was then determined using the Fiji software per the software instructions. In cell viability experiments, viable and dead cells were manually counted. Viability was calculated as the percentage of viable cells relative to the total number of cells. In cell stress determinations, we quantified the area occupied by CellEvent positive cells and normalised to the area occupied by Hoechst33342 staining. ROS increase ratio was calculated as CellROX fluorescence intensity for the harvested and reseeded cells normalised to the fluorescence intensity of control cells.

### Statistical analysis

All the experiments were repeated at least three times as independent biological repeats. All results are presented as the mean ± standard error. The normal distribution was challenged by the Kolmogorov-Smirnov test. Statistical significance was set at p < 0.05. Multiple comparisons by ANOVA were corrected using the Holm–Sidak test. Where the assumptions of one-way ANOVA were violated, the non-parametric Kruskal–Wallis test was performed followed by Dunn’s multiple comparison tests.

## Supplementary information

Supplementary video S1

Supplementary video S2

Supplementary info
