# Author Correction: Enabling cell recovery from 3D cell culture microfluidic devices for tumour microenvironment biomarker profiling

**DOI:** 10.1038/s41598-020-57830-0

**Published:** 2020-01-15

**Authors:** María Virumbrales-Muñoz, Jose M. Ayuso, Alodia Lacueva, Teodora Randelovic, Megan K. Livingston, David J. Beebe, Sara Oliván, Desirée Pereboom, Manuel Doblare, Luis Fernández, Ignacio Ochoa

**Affiliations:** 1 Department of Biomedical Engineering, Wisconsin Institutes for Medical Research, University of Wisconsin-Madison, 1111 Highland Avenue, Madison, Wisconsin 53705 United States; 2 Medical engineering, Morgridge Institute for Research, 330 N Orchard street, Madison, WI 53715 USA; 3 Group of Applied Mechanics and Bioengineering (AMB), Aragón Institute of Engineering Research (I3A), University of Zaragoza, Zaragoza, Spain; 4 Centro Investigacion Biomedica en Red. Bioingenieria, biomateriales y nanomedicina (CIBER-BBN), Madrid, Spain; 5 Aragon Institute for Health Research (IIS Aragón), Instituto de Salud Carlos III, Zaragoza, Spain; 6 Department of Chemistry, University of Wisconsin-Madison, Madison, USA; 7 Department of Pathology and Laboratory Medicine, University of Wisconsin-Madison, 1111 Highland Avenue, Madison, Wisconsin 53705 United States; 8 Servicio General de Apoyo a la Investigación de Citómica, University of Zaragoza, Zaragoza, Spain

Correction to: *Scientific Reports* 10.1038/s41598-019-42529-8, published online 17 April 2019

The Acknowledgements section in this Article is incomplete.

“Authors would like to thank Dr Edorta Santos for assistance on hydrogel degradation conditions, Inés Mármol for enriching discussions on the qPCR experiments and Jacobo Ayensa for valuable advice on statistical analysis. This work has been supported by the Spanish national research program (DPI2015-65401-C3-1-R and BIO2016-79092-R grants) and the Aragon Government (T24_17R grant). The Spanish national government provided M.V. studentship. Authors would like to acknowledge the use of Servicio General de Apoyo a la Investigación-SAI, Universidad de Zaragoza. ORCHID and CISTEM projects have received funding from H2020 programme under grant agreement 766884 and 778354 respectively.”

should read:

“Authors would like to thank Dr Edorta Santos for assistance on hydrogel degradation conditions, Inés Mármol for enriching discussions on the qPCR experiments and Jacobo Ayensa for valuable advice on statistical analysis. This work has been supported by the Spanish national research program (DPI2015-65401-C3-1-R and BIO2016-79092-R grants) and the Aragon Government (T24_17R grant). The Spanish national government provided M.V. studentship. Authors would like to acknowledge the use of Servicio General de Apoyo a la Investigación-SAI, Universidad de Zaragoza. ORCHID and CISTEM projects have received funding from H2020 programme under grant agreement 766884 and 778354 respectively. M.V., D.J.B and M.K.L. acknowledge University of Wisconsin Carbone Cancer Center Support Grant P30 CA014520, Environmental Protection Agency 83573701-Human Models for Analysis of Pathways (H-MAPs) Center and NIH NCI Cancer Moonshot Grant R33CA225281.”

