# Supplementary material for: Enabling cell recovery from 3D cell culture microfluidic devices for tumour microenvironment biomarker profiling
Source: Sci Rep. 2019 Apr 17;9:6199. doi: 10.1038/s41598-019-42529-8 (PMC6470149; doi:10.1038/s41598-019-42529-8)
Supplement: Supplementary file 3 — Supplementary info [file 41598_2019_42529_MOESM3_ESM.docx]

**Enabling cell recovery from 3D cell culture microfluidic devices for tumour microenvironment biomarker profiling.**

Maria Virumbrales-Muñoz^1^, Jose M. Ayuso^1,2^, Alodia Lacueva^3,4,5^, Teodora Randelovic^3,4,5^, Megan K. Livingston^1^, David J. Beebe^1,6^, Sara Oliván^3,4,5^, Desirée Pereboom^7^, Manuel Doblare^3,4,5^, Luis Fernández^3,4,5,*^, Ignacio Ochoa^3,4,5, *^.

1. Department of Biomedical Engineering, Wisconsin Institutes for Medical Research, University of Wisconsin-Madison, 1111 Highland Avenue, Madison, Wisconsin 53705, United States.

2. Medical Engineering, Morgridge Institute for Research, 330 N Orchard street, Madison, WI, USA. 53715.

3. Group of Applied Mechanics and Bioengineering (AMB), Aragón Institute of Engineering Research (I3A), University of Zaragoza, Spain.

4. Centro Investigacion Biomedica en Red. Bioingenieria, biomateriales y nanomedicina (CIBER-BBN), Spain.

5. Aragon Institute for Health Research (IIS Aragón), Instituto de Salud Carlos III, Spain.

6. Department of Pathology and Laboratory Medicine. University of Wisconsin, Madison, 1111 Highland Avenue, Madison, Wisconsin 53705, United States.

7. Servicio General de Apoyo a la Investigación de Citómica. University of Zaragoza, Spain.

*Authors contributed to the work equally.

# Supplementary information

**Supplementary Video S1: Time-lapse confocal reflection microscopy of hydrogel degradation when treated with a 2 mg/ml collagenase solution.** Collagen fibres are shown in blue, and they disappear over time due to collagenase degradation.

**Supplementary Video S2: Time-lapse of cell viability during hydrogel degradation when treated with an 8 mg/ml collagenase solution.** Cells are tracked with CMFDA tracker (green), and dead cells are stained with propidium iodide in real time (red).

**Table S1: Linear fitting parameters for the characterisation of the degradation kinetics of collagen via enzymatic degradation.**

| **Fitted data** | **N** | **Slope (average ± SD)** | **Y intercept  (average ± SD)** | **R^2^** |
| --- | --- | --- | --- | --- |
| **Linear region of collagen degradation dynamic with 0.5 mg/ml collagenase (Fig. 3b)** | 564 | -0.0881 ± 0.000165 | 107 ± 0.119 | 0.998 |
| **Linear region of collagen degradation dynamic with 2 mg/ml collagenase (Fig. 3b)** | 71 | -0.377 ± 0.00449 | 111 ± 0.728 | 0.990 |
| **Linear region of collagen degradation dynamic with 8 mg/ml collagenase (Fig. 3b)** | 28 | 1.03 ± 0.0145 | 123 ± 1.04 | 0.995 |
| **Velocity of degradation for each collagenase concentration (Fig. 3c)** | 663 | 0.130 ± 0.0007645 | 0.03153 ± 0.00140 | 0.978 |

**Table S2: RT-PCR primers for the chosen targets and reference genes for gene profiling of the tumour microenvironment.** Sequences are detailed for both forward and reverse primers.

| **Primer list** | | |
| --- | --- | --- |
| Gene name | Primers | Exon location |
| *Gapdh*  (Reference) | 5’-TGTAGTTGAGGTCAATGAAGGG-3’  5’-ACATCGCTCAGACACCATG-3’ | 2-3 |
| *Actb* (Reference) | 5’- CCTTGCACATGCCGGAG-3’  5’- ACAGAGCCTCGCCTTTG-3’ | 1-2 |
| *Ralbp1* | 5’-TCATCTCCAGAGCTATCTTCCT-3’  5’-CATGAGCCTCCTGATGTAGTG-5’ | 2-3 |
| *Mki67* | 5’- GAAGCTGGATACGGATGTCA-3’  5’- CGCCTGGTTACTATCAAAAGGA-3’ | 2-3 |
| *Slc2a1* | 5’-GGCCACAAACGGAAAGATG-3’  5’-GTGCCATACTCATGACCATCG-5’ | 8-9 |

**
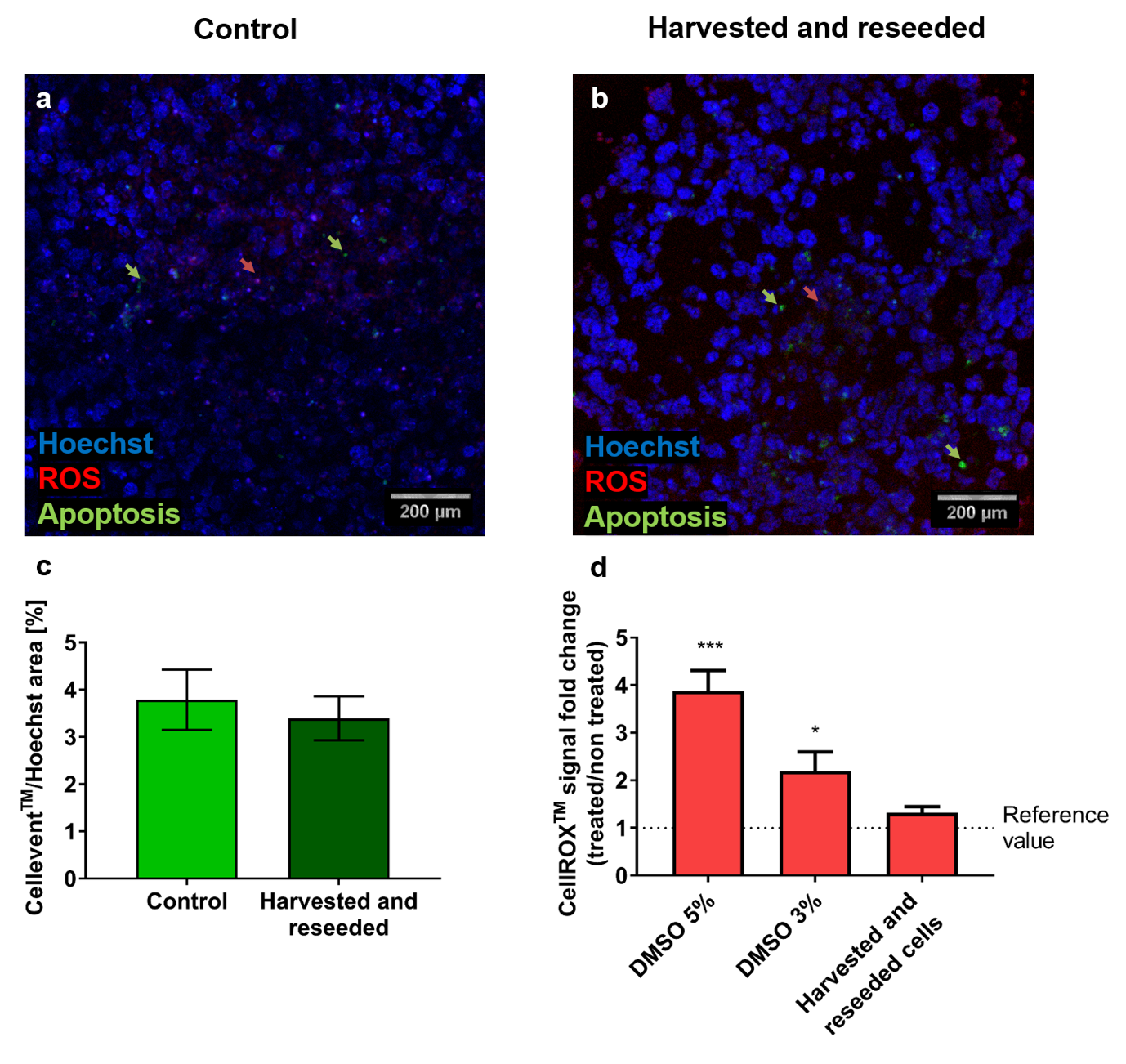
**

**Supplementary figure S1: Cell stress response induction of the cell extraction method. a)** First, we embedded HCT-116 cells in collagen hydrogels. After 24 hours, cells were extracted from the hydrogels by enzymatic degradation with an 8 mg/ml solution of collagenase in PBS. Recovered cells were washed with PBS and re-embedded in collagen hydrogels. Cell viability was assessed after 72 hours using confocal imaging, the cell nuclei were stained (Hoechst 33342, blue), a cell stress indicator was added to distinguish dead cells (CellROX, red), as well as an early apoptosis indicator (CellEvent, green). Results from extracted reseeded cells were compared with non-treated cells (control). Images of the control cells can be observed in **(a)** and images of harvested and reseeded cells can be observed in **(b)**. **(c)** CellEvent-positive cells were normalized to total nuclei in area and are expressed as percentage. Reseeded cells were found non-significantly different to control cells (p=0.6225, Samples passed Shapiro-Wilk normality test and were subjected to Student’s t test with Welch correction). **(d)** CellROX intensity fold change was quantified for harvested and reseeded cells and normalized to control cells. CellROX intensity fold change in the samples was found to be non-significantly different from the reference value (p = 0.9215), unlike positive controls with 3% or 5% of DMSO (p=0.0521 and p<0.0001, respectively as assessed via one-way ANOVA and Tukey post-hoc test after passing Shapiro-Wilk normality test).


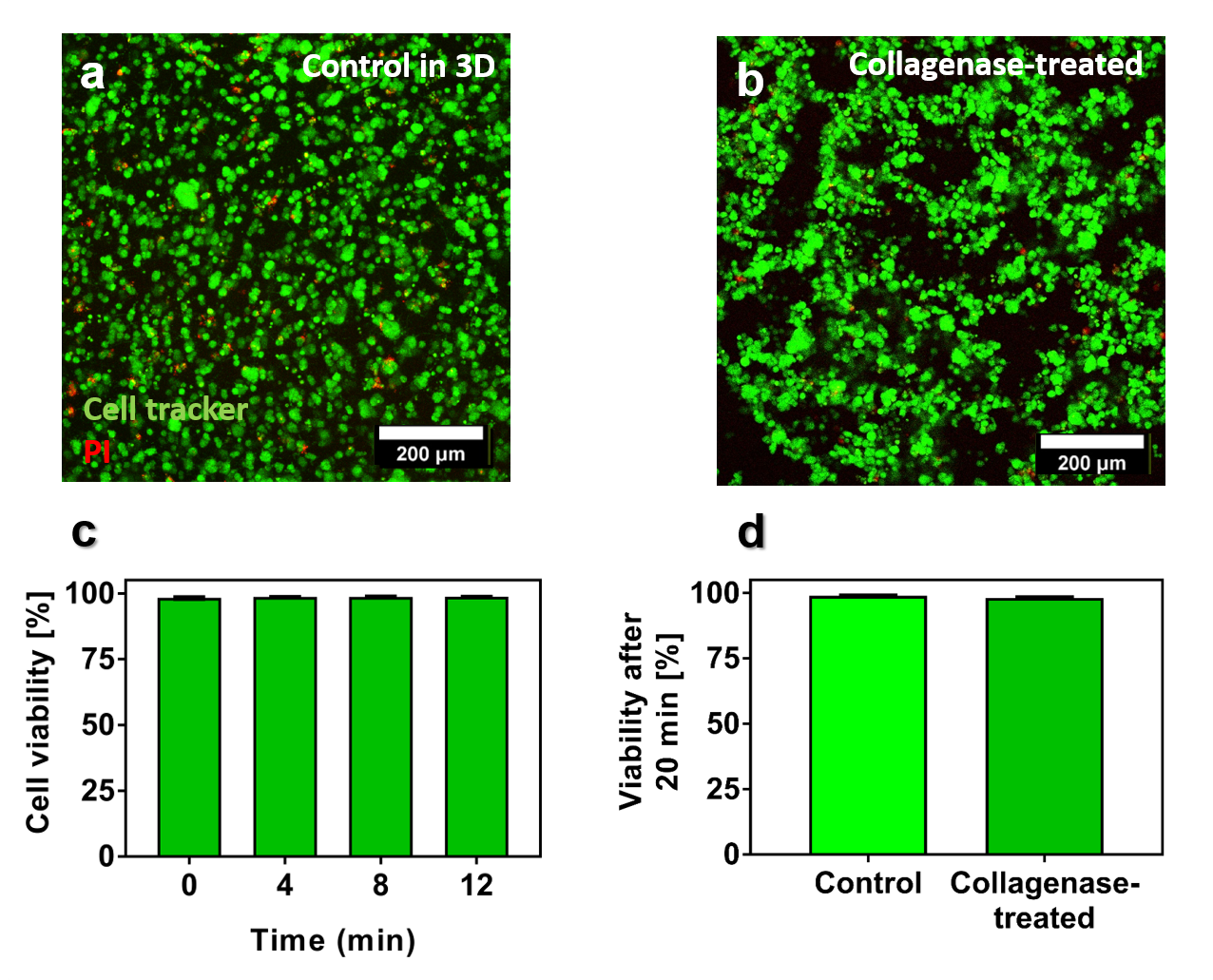


**Supplementary Figure S2: Real-time tracking of cell viability during the collagenase degradation procedure.** Cells were stained with cell tracker and propidium iodide and embedded in a 2 mg/ml hydrogel. **(a)** A control hydrogel and **(b)** an 8 mg/ml collagenase-treated hydrogel were imaged for a period of 20 minutes. **(c)** cell viability was analyzed for the degrading hydrogel at 0, 4, 8 and 12 minutes. (non-significant via one-way ANOVA and post-hoc Bonferroni test) **(d)** Cell viability was compared between the control and collagenase-treated hydrogels after 20 minutes of collagenase addition. (non-significant via Students’ t-test). Samples passed Shapiro Wilk’s normality test. N= 3.

**
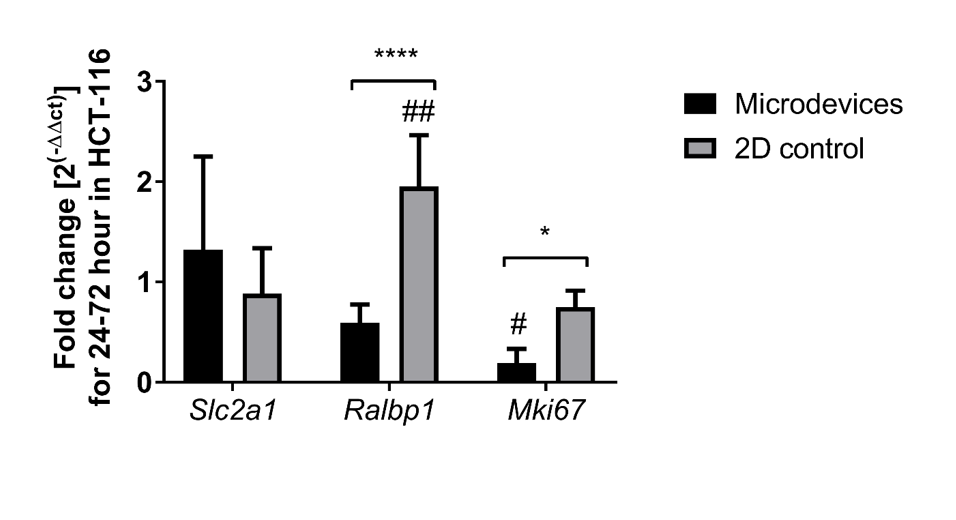
**

**Supplementary figure S3:** **Comparison of gene expression between microdevices and 2D controls.** qPCR analysis of extracted RNA of microdevices and 2D controls. Expression fold changes in *Slc2a1* (Glut-1), *Ralbp1* (ralA binding protein 1) and *Mki67* (Ki-67) were assessed between 24 and 72h in samples recovered from the microdevice with the described method as well as 2D controls. *Actb* and *Gapdh* were used as reference (housekeeping) genes for normalisation. *Ralbp1* and *Mki67* yielded significantly different results in microdevices and 2D controls (p<0.0001 and p = 0.012, respectively). Only Ralbp1 2D control was significantly different from the reference value (p= 0.0211). Samples passed Shapiro-Wilk normality test and were subjected to two-way ANOVA with FDR method of Benjamini, Krieger and Yekutieli.


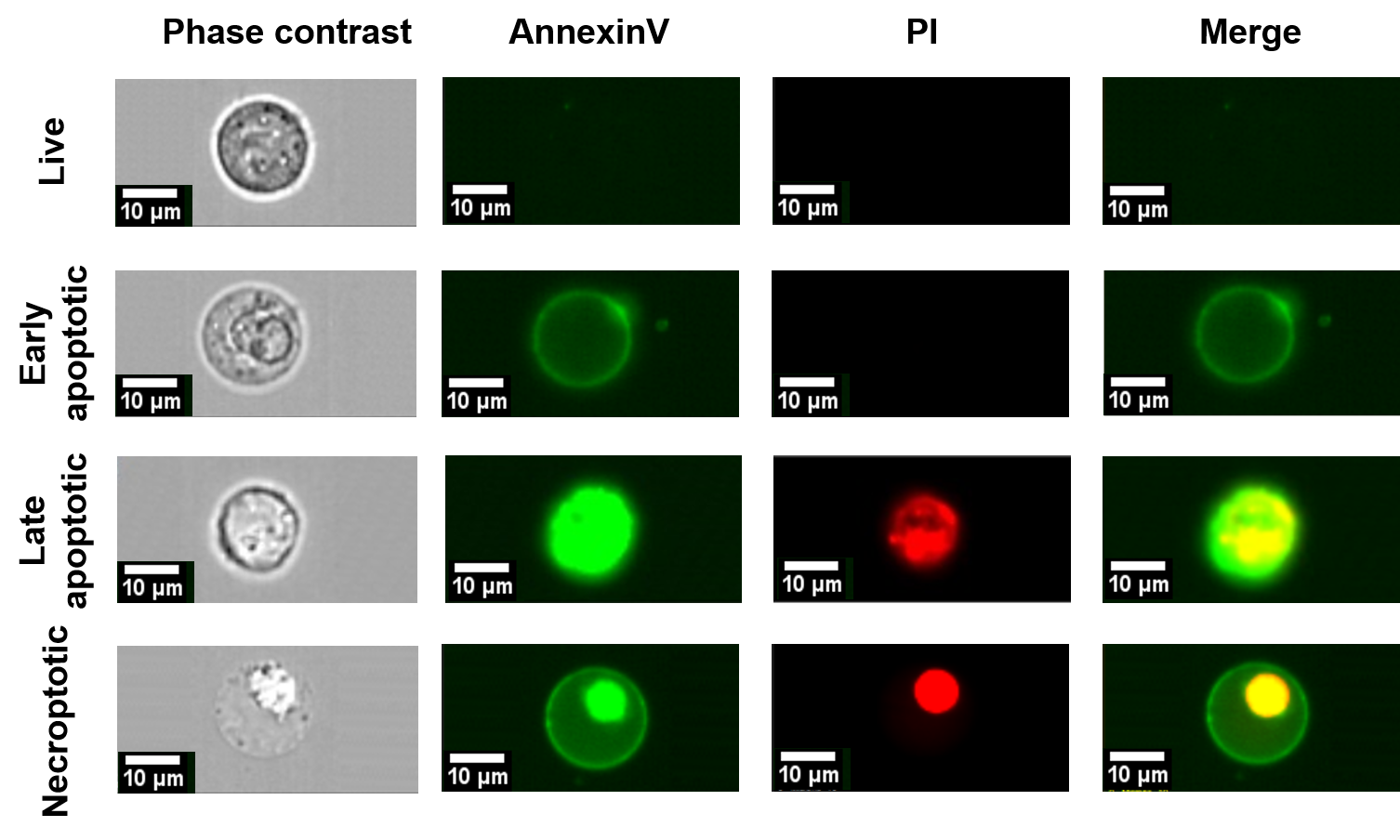
**Supplementary Figure S4: Sample image of AMNIS® flow cytometry fluorescence channels and phase contrast for the cell viability and death study.** Images designated live cell (AnnexinV^-^  / PI^-^) , early apoptotic (AnnexinV^+^ / PI^-^), late apoptotic cell (AnnexinV^+^ / PI^+^ and inhomogeneous nucleus) and necroptotic (AnnexinV^+^ / PI^+^ and intact nucleus) are included. A sample image of necrotic cell is not included because this population size was negligible in all cases.

# Supplementary results

To expand on the cell recovery protocol described in this article and provide insight into cell recovery for other device architectures, we decided to include more device geometries in our paper. Hence, we studied the degradation of collagen type I hydrogels in three additional device architectures to that used in this article. Within these architectures, we also tested two concentrations of collagen I: 2 and 6 mg/mL[^1-3^](#_ENREF_1). We chose to use these concentrations because they are widely used in *in vitro* cell culture studies. Likewise, lower collagen concentrations do not support lumen formation in some devices[^4^](#_ENREF_4).

To track hydrogel degradation and particle recovery, we added Fluospheres to the hydrogel mixture (as described for Figure 4) and 10% of the collagen was substituted for fluorescently-conjugated collagen. We incubated the hydrogels with the 8 mg/ml (18 IU) collagenase mixture and tracked the degradation of the hydrogels based on the release of fluorescent beads and depletion of fluorescent collagen signal in different device architectures:

First, we used LumeNEXT^[4](#_ENREF_4)^, a device used to recapitulate ductal structures was fabricated using soft-lithography (Fig. S5a). The device was filled with the hydrogel and bead mixture (Fig. S5b). After polymerization, a sacrificial tubular PDMS rod was removed to create a duct-like structure in the hydrogel. During the collagen degradation studies, collagenase was introduced to the system through the duct-like structure, rapidly diffusing into the hydrogel (Fig. S5c-d). Interestingly, over 80% of the recovery happened in the first 12 minutes of incubation for the 2 mg/ml hydrogel-filled devices (remaining areas were 13.58 ± 8.078 for the hydrogel and 16.35 ± 5.878 for the beads), whereas the 6 mg/ml hydrogel-filled devices took 20 minutes to get similar percentages (Fig S5e-f). In both cases, a final rinse after the 20-minute incubation yielded minimal remaining hydrogel areas. Specifically, the remainder of hydrogel in the region of interest analysed for the 2 mg/ml collagen-filled devices was 0.403 ± 0.192 %, whereas the remainder of beads was 1.27 ± 0.289 %. On the other hand, the remainder of hydrogel for the collagen in the 6 mg/ml collagen-filled devices was 0.62 ± 0.62 %, and the remainder of beads was 4.15 ± 2.89 % (Fig S5e-f). While the curves resulting from the quantification of fluorescent collagen at 2 mg/ml and 6 mg/ml were not different from each other, the difference between the curves resulting from bead quantification was statistically significant (p = 0.031) (Fig S5e-f).


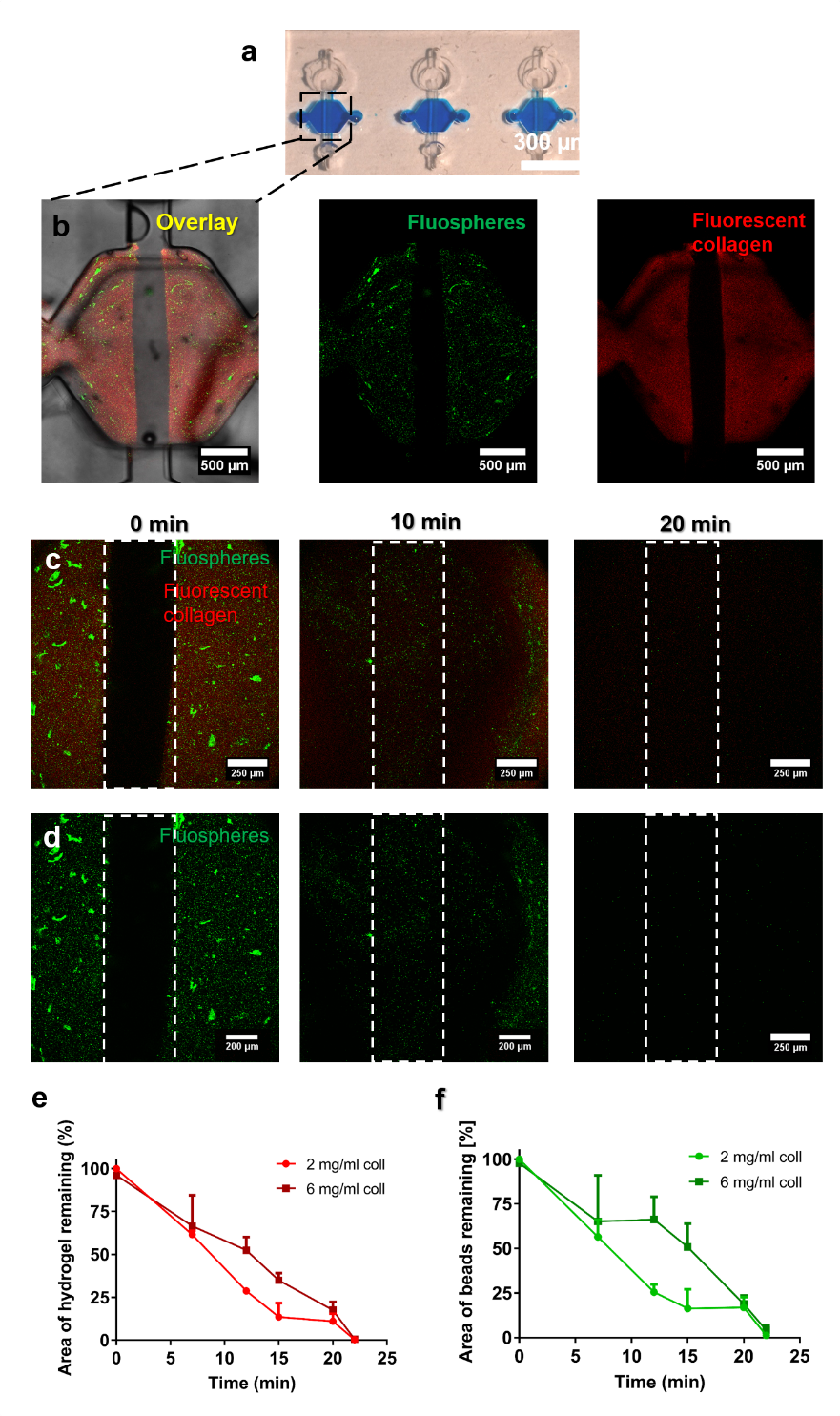


**Supplementary Figure S5: Recovery efficiency study in LumeNEXT device. (a)** Photograph of the LumeNEXT microdevices filled with blue dye for visualisation purposes. **(b)** Phase contrast image (greyscale) of device filled with 2 mg/ml collagen supplemented with Fluospheres (depicted in green) and 10% of Alexa 488 collagen (depicted in red) of the final concentration. **(c)** Composite image of collagen (green) and fluospheres (red) at time 0, 10 and 20 minutes of incubation with collagenase solution. **(d)** Fluosphere channel of (c), for better visualization of the Fluospheres. **(e)** The area with remaining fluorescent collagen was quantified for the 2 mg/ml and 6 mg/ml collagen at different time points in the collagenase degradation procedure within the device chamber. **(f)** The same quantification was performed for Fluospheres on the 2 mg/ml and 6 mg/ml collagen hydrogels.

The second device tested was a soft lithography-fabricated co-culture channel device[^5^](#_ENREF_5), in which the two culture channels are separated by rectangular cross-section diffusion channels on the bottom of the channels (Fig. S6a). The hydrogel mixture was injected in one of the culture channels (left), whereas the other channel was used for introduction of the collagenase (right channel, Fig S6b). The hydrogel was pinned in the left channel and filled the diffusion channels. The collagenase solution was only in contact with the hydrogel through the diffusion channels, and therefore had to diffuse through to get to the hydrogel, slowing down the degradation process (Fig S6c-d).

In the co-culture channel device, over 80% of the recovery happened in the first 15 minutes of incubation for all devices, independently of collagen concentration. The remaining areas were 18.81 ± 9.387 % for collagen and 16.35 ± 5.878 % for the beads in the 2 mg/ml hydrogel-filled devices; 15.11 ± 12.703 % for collagen and 11.54 ± 6.72 % in the 6 mg/ml hydrogel filled devices. In this case, the variability between different experiments was higher for the 6 mg/ml devices than for the 2 mg/ml collagen, but the resulting curves were not significantly different from each other.

After the final rinse, the remainder of hydrogel in the region of interest analysed for the 2 mg/ml collagen-filled devices was 5.97 ± 2.64 %, whereas the remainder of beads was 5.50 ± 3.21 %. On the other hand, the remainder of hydrogel for the collagen in the 6 mg/ml collagen-filled devices was 0.69 ± 0.17 %, and the remainder of beads was 3.521 ± 2.42 %.


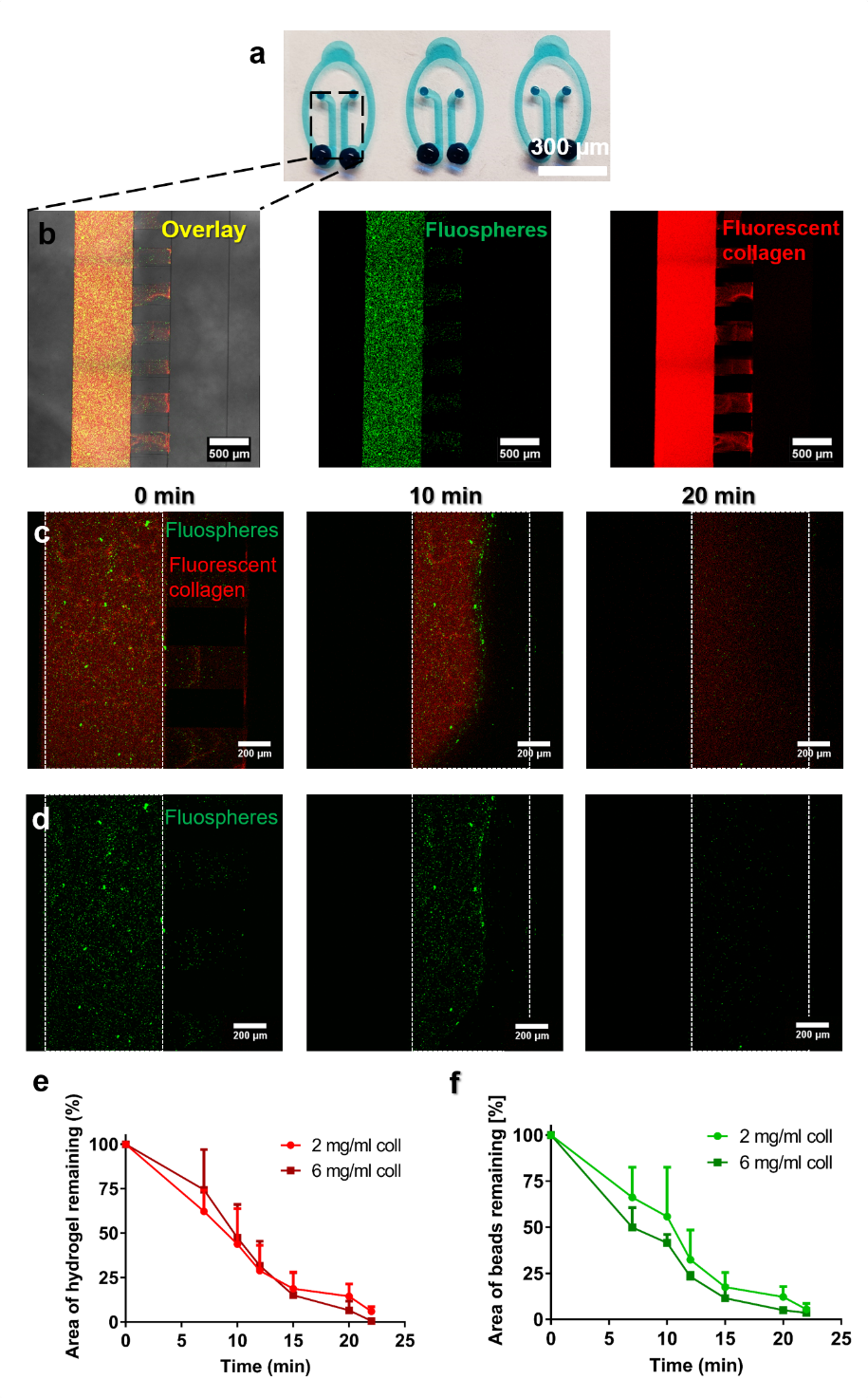


**Supplementary Figure S6: Recovery efficiency study in the co-culture diffusion device. (a)** Photograph of the co-culture microdevices filled with blue dye for visualisation purposes. **(b)** Phase contrast image (greyscale) of device filled with 2 mg/ml collagen supplemented with Fluospheres (depicted in green) and 10% of Alexa 488 collagen (depicted in red) of the final concentration. **(c)** Composite image of collagen (green) and Fluospheres (red) at time 0, 10 and 20 minutes of incubation with collagenase solution. **(d)** Fluosphere channel of (c), for better visualization of the Fluospheres. **(e)** The area with remaining fluorescent collagen was quantified for the 2 mg/ml and 6 mg/ml collagen at different time points in the collagenase degradation procedure within the device chamber. **(f)** The same quantification was performed for Fluospheres on the 2 mg/ml and 6 mg/ml collagen hydrogels.

The third device tested is a soft-lithography-based tri-channel device[^6^](#_ENREF_6), of most similar architecture to the gradient device used in the main text of this manuscript (Fig S7a). Therefore, the hydrogel mixture was also injected in the central channel and allowed to polymerize (Fig S7b). Next, the collagenase solution was injected through the two flanking channels, and collagenase had contact with the hydrogel through the diffusion channels (Fig 7c-d).

In the tri-channel device, after 15 minutes of incubation the remaining areas were 17.3 ± 13.2 % for collagen and 32.9 ± 24.6 % for the beads in the 2 mg/ml hydrogel-filled devices; 47.8 ± 0.84% for collagen and 48.9 ± 3.12 % in the 6 mg/ml hydrogel filled devices. We observed that the final area occupied by collagen and beads had a small error bar, indicating that the recovery was consistent among the studied devices (Fig 7e-f).

After the final rinse, the remainder of hydrogel in the region of interest analysed for the 2 mg/ml collagen-filled devices was 1.15 ± 0.461 %, whereas the remainder of beads was 6.35 ± 3.00 %. On the other hand, the remainder of hydrogel for the collagen in the 6 mg/ml collagen-filled devices was 19.96 ± 0.786 %, and the remainder of beads was 19.19 ± 2.12 %. Although this recovery was the lowest of all the conditions tested, recoveries are still over 80% (Fig 7e-f).


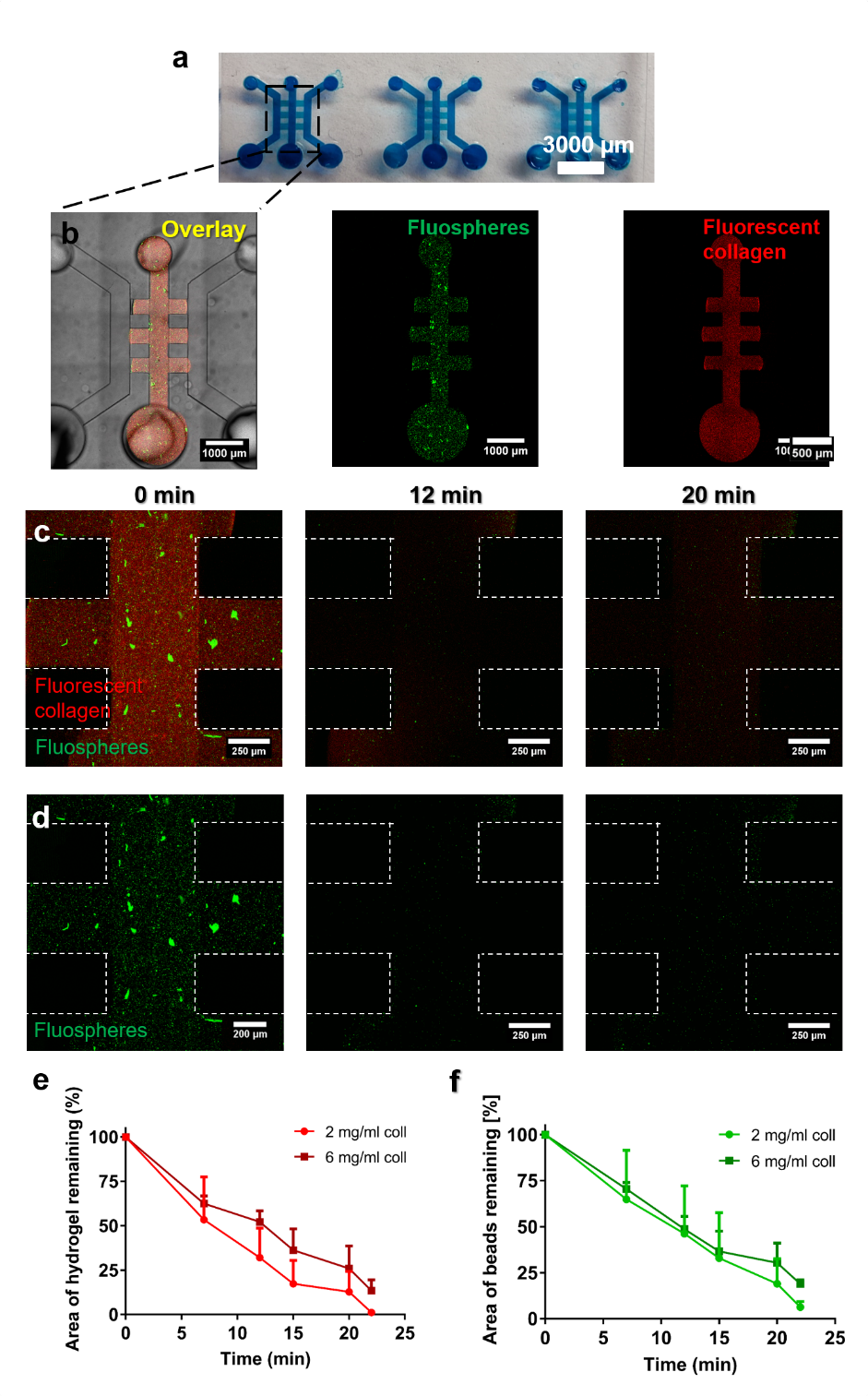


**Supplementary Figure S7: Recovery efficiency study in the tri-channel device.** **(a)** Photograph of the tri-channel microdevices filled with blue dye for visualisation purposes. **(b)** Phase contrast image (greyscale) of device filled with 2 mg/ml collagen supplemented with Fluospheres (depicted in green) and 10% of Alexa 488 collagen (depicted in red) of the final concentration. **(c)** Composite image of collagen (green) and fluospheres (red) at time 0, 10 and 20 minutes of incubation with collagenase solution. **(d)** Fluosphere channel of (c), for better visualization of the Fluospheres. **(e)** The area with remaining fluorescent collagen was quantified for the 2 mg/ml and 6 mg/ml collagen at different time points in the collagenase degradation procedure within the device chamber. **(f)** The same quantification was performed for Fluospheres on the 2 mg/ml and 6 mg/ml collagen hydrogels.

References

1 Antoine, E. E., Vlachos, P. P. & Rylander, M. N. Review of collagen I hydrogels for bioengineered tissue microenvironments: characterization of mechanics, structure, and transport. *Tissue Engineering Part B: Reviews* **20**, 683-696 (2014).

2 Bischel, L. L. *et al.* The importance of being a lumen. *The FASEB Journal* **28**, 4583-4590 (2014).

3 Hui, T., Cheung, K., Cheung, W., Chan, D. & Chan, B. In vitro chondrogenic differentiation of human mesenchymal stem cells in collagen microspheres: influence of cell seeding density and collagen concentration. *Biomaterials* **29**, 3201-3212 (2008).

4 Jiménez‐Torres, J. A., Peery, S. L., Sung, K. E. & Beebe, D. J. LumeNEXT: a practical method to pattern luminal structures in ECM gels. *Advanced healthcare materials* **5**, 198-204 (2016).

5 Lang, J. D., Berry, S. M., Powers, G. L., Beebe, D. J. & Alarid, E. T. Hormonally responsive breast cancer cells in a microfluidic co-culture model as a sensor of microenvironmental activity. *Integr Biol (Camb)* **5**, 807-816, doi:10.1039/c3ib20265h (2013).

6 Bischel, L. L., Young, E. W., Mader, B. R. & Beebe, D. J. Tubeless microfluidic angiogenesis assay with three-dimensional endothelial-lined microvessels. *Biomaterials* **34**, 1471-1477 (2013).
